# Context Matters—Why We Need to Change From a One Size Fits all Approach to Made-to-Measure Therapies for Individual Patients With Pancreatic Cancer

**DOI:** 10.3389/fcell.2021.760705

**Published:** 2021-11-04

**Authors:** Sushmitha Sankarasubramanian, Ulrike Pfohl, Christian R. A. Regenbrecht, Christoph Reinhard, Lena Wedeken

**Affiliations:** ^1^ CELLphenomics GmbH, Berlin, Germany; ^2^ Department of Surgery, Universitätsklinikum Erlangen, Erlangen, Germany; ^3^ ASC Oncology GmbH, Berlin, Germany; ^4^ Institute for Molecular Bio Science, Goethe University Frankfurt Am Main, Frankfurt, Germany; ^5^ Institute for Pathology, Universitätsklinikum Göttingen, Göttingen, Germany

**Keywords:** pancreatic ductal adenocarcinoma (PDAC), tumor heterogeneity, KRAS, 3D cell culture models, personalized medicine, patient-derived tumor organoids, combined targeted and phenotypic approach, reverse clinical engineering

## Abstract

Pancreatic cancer is one of the deadliest cancers and remains a major unsolved health problem. While pancreatic ductal adenocarcinoma (PDAC) is associated with driver mutations in only four major genes (*KRAS, TP53, SMAD4,* and *CDKN2A*), every tumor differs in its molecular landscape, histology, and prognosis. It is crucial to understand and consider these differences to be able to tailor treatment regimens specific to the vulnerabilities of the individual tumor to enhance patient outcome. This review focuses on the heterogeneity of pancreatic tumor cells and how in addition to genetic alterations, the subsequent dysregulation of multiple signaling cascades at various levels, epigenetic and metabolic factors contribute to the oncogenesis of PDAC and compensate for each other in driving cancer progression if one is tackled by a therapeutic approach. This implicates that besides the need for new combinatorial therapies for PDAC, a personalized approach for treating this highly complex cancer is required. A strategy that combines both a target-based and phenotypic approach to identify an effective treatment, like Reverse Clinical Engineering^®^ using patient-derived organoids, is discussed as a promising way forward in the field of personalized medicine to tackle this deadly disease.

## Introduction

Treating pancreatic cancer is a major clinical challenge. It is aggressive, often diagnosed late in its course and treatment options are not only limited but also with a low success rate—despite strong efforts in basic and clinical research to better understand and tackle this deadly disease.

The most frequent histological type of pancreatic cancer is the pancreatic ductal adenocarcinoma (PDAC) arising from epithelial ductal cells of the pancreas (Warshaw and Castillo, 1992; Li et al., 2004). PDAC is among the cancers with the worst prognosis with a 5-year survival rate of less than 9% (Siegel et al., 2021) and is predicted to be the second leading cause of cancer death by 2030 (Rahib et al., 2014).

The only curative treatment for PDAC so far is surgery, but most of the patients are diagnosed at late stages and already metastasized. 85% of PDACs are unresectable (Seufferlein et al., 2014; Orth et al., 2019) and currently the most common treatment for these patients is chemotherapy that includes combinations with gemcitabine and 5-fluorouracil (5-FU). Combination therapy of gemcitabine and nab-paclitaxel improved overall survival to gemcitabine therapy alone by 1.8 months (8.5 vs 6.7 months median overall survival) with the 2-years survival rate increasing to 9% on gemcitabine plus nab-paclitaxel compared to 4% on gemcitabine therapy alone (Von Hoff et al., 2013; Saito et al., 2017).

Improved therapy results were also shown for FOLFIRINOX, which is a combination of 5-FU, irinotecan, oxaliplatin, and folinic acid, but also exhibits increased side effects and affects the quality of life (Conroy et al., 2011). For patients treated with FOLFIRINOX, the overall survival increased by 4.3 months compared to gemcitabine (11.1 vs 6.8 months) (Conroy et al., 2011), and the response rate to gemcitabine or FOLFIRINOX therapy was only 10 and 31% respectively (Conroy et al., 2011; Bian et al., 2017).

While these two regimes were considered as a success story in the therapeutic arena of PDAC, the overall survival is still very low with only small improvements, illustrating that an effective treatment of PDAC is still missing. Further, chemoresistance of the tumor is prevalent and is one of the main reasons for the very low survival rate of this aggressive cancer (Juiz et al., 2019).

Targeted therapies aiming specifically at genomic aberrant pathways, often using specific molecular profiles of individual cancer to stratify patients, significantly enhanced cancer treatment—but not yet for PDAC. For example, colorectal cancer patients with wild type proto-oncogene *KRAS* or *BRAF* benefit often from treatment with monoclonal antibodies targeting the epidermal growth factor receptor (EGFR) (Amado et al., 2008; Karapetis et al., 2008; Di Nicolantonio et al., 2008). In PDAC, combination therapy of the anti-EGFR antibody erlotinib and gemcitabine has been approved as a first line therapy for metastatic disease, independent of *KRAS* mutational status, and showed clinical benefit compared to gemcitabine alone (Moore et al., 2007; Wang et al., 2015). However, also here the median survival time increased only to 6.24 months compared to 5.91 months for gemcitabine treatment alone in the initial trial (Moore et al., 2007) and *KRAS* mutational status was shown as not predictive for the treatment response to erlotinib in PDAC (Boeck et al., 2013).

In PDAC, some targeted therapies improved treatment but unfortunately only for a small proportion of patients with specific aberrations. PDAC patients with high microsatellite instability or DNA mismatch repair-deficient tumors (0.8% of PDAC cases) were shown to respond to immune checkpoint blockade inhibitors, such as anti PD-1/PD-L1, with durable responses (Diaz et al., 2017; Hu et al., 2018; Eso et al., 2020). Therapies targeting either DNA damage repair (i.e., PARP inhibitors) in germline *BRCA* mutated metastatic PDAC (about 7% of patients) (Vincent et al., 2011a; Golan et al., 2019) and therapies targeting specific oncogenes such as mutant BRAF (about 4% of patients) or kinase fusion genes in *KRAS* wild type tumors (about 4% of patients) (Luchini et al., 2020) were also shown to benefit patients in a clinically relevant fashion.

So why is there so little progress in PDAC treatment? The highly desmoplastic tumor stroma (Öhlund et al., 2017; Hosein et al., 2020) making it difficult for a drug to reach the tumor is considered as one reason for treatment failure in PDAC (not reviewed here). Another is the effective immune-evasion mechanisms employed by PDAC (Saka et al., 2020) (not reviewed here). But on the other hand, it is the individual differences at the molecular and cellular level of each tumor, its heterogeneity, as well as the interplay between various pathways and dysregulations, the context that confers different susceptibility to drugs. These tumor-cell intrinsic features are the topic of this review.

Much is known about the genetic landscape and mutations driving PDAC development and progression. PDAC is associated with mutations in only four major genes: the proto-oncogene *KRAS* as the main disease driver, followed by tumor suppressor genes tumor protein 53 (*TP53*), SMAD family member 4 (*SMAD4*) and cyclin dependent kinase inhibitor 2A (*CDKN2A*) (Hingorani et al., 2005). The current model for PDAC development is that genomic alterations occur in a stepwise manner with mutations in *KRAS* and *CDKN2A* preceding in early Pan-IN lesions, which are precursor lesions for invasive carcinoma, often followed by mutations in *TP53* and *SMAD4* which contribute to the tumor’s invasiveness (Aguirre et al., 2003; Hingorani et al., 2005; Iacobuzio-Donahue et al., 2012; Ryan et al., 2014).

However, despite overwhelming research on PDAC biology and genetics, we were not yet able to harness this knowledge to develop effective targeted therapies for pancreatic cancer. The main lingering question that remains is how to translate the knowledge of disease biology and its heterogeneity into targeted therapies for individual patients. It is much needed to unearth the tumors’ resistance mechanisms and identify chemo-sensitivity signatures in PDAC, which will increase the chances of identifying clinically effective targeted therapies.

This review delineates the difficulties in targeting highly heterogenous, mutant KRAS-driven PDAC cells due to the presence of dysregulations at multiple level. Dysregulations at genome level is just one aspect driving this disease but there are also dysregulations at the metabolic space, epigenetic alterations and additional pathway deregulations that enhance tumor progression. In addition, several compensatory mechanisms are utilized by PDAC cells for their survival when the highly dysregulated KRAS signaling pathway is targeted. Therefore, identifying and targeting vulnerabilities not only in the genetic landscape but also at the epigenetic and metabolic level is necessary for effective treatment. Further, it is required to approach PDAC therapy in an individualized manner to take the heterogeneity and the context of the multiple dysregulations into account. This review further highlights that patient-derived organoids are an excellent tool to functionally profile individual tumors and that Reverse Clinical Engineering^®^, an approach that combines target-based and phenotypic screening strategies as a single system in a potentially high-throughput manner holds an important component of the future of personalized oncology.

## Distinct Subtypes Reveal PDAC Heterogeneity

Molecular taxonomy of PDAC has been described in several gene expression studies in different, independent cohorts. Collisson and colleagues identified three subtypes (quasi mesenchymal, classical, and exocrine-like) in 2011, based on analyses of global gene expression from human and mice PDAC lines along with micro-dissected human PDAC tissue and found that these subtypes progress and respond to treatment differently (Collisson et al., 2011). A 2015 study by Moffitt et al. on bulk resected primary, non-treated PDAC tumors and metastases stratified PDAC into tumor-specific and stromal-specific subtypes using a sophisticated computational approach (Moffitt et al., 2015). Distinct molecular mechanisms associated with distinct tumor subtypes were identified by the Australian Pancreatic Cancer Genome Initiative in 2016 using integrated genomic, epigenomic, and transcriptomic analysis. Here, four subtypes were identified based on whole genome, deep exome, and RNA expression profiles: squamous, pancreatic progenitor, immunogenic and aberrantly differentiated exocrine- and endocrine-like (Bailey et al., 2016). Differential expression of transcription factors and their downstream effectors among these subtypes revealed the heterogeneity in PDAC pathophysiology.

Distinct classifications from different cohorts (Collisson et al., 2011; Moffitt et al., 2015; Bailey et al., 2016; Puleo et al., 2018) overlap (Raphael et al., 2017) and altogether sum up to two major tumor-specific subtypes based on high purity tumor samples: classical/pancreatic progenitor and squamous/basal-like. Both subtypes are associated with specific genetic programs and prognosis. The classical subtype has a higher level of differentiation and better prognosis compared to the basal-like/squamous subtype (Bailey et al., 2016).

A study from 2020 further improved the purification of PDAC tumor cells for genomic analysis using laser capture microdissection and classified the two major PDAC subtypes into further subclasses based on the degree of squamous signatures and clinical staging to classical-like A, classical-like B, basal-like A, basal-like B, and hybrid (Chan-Seng-Yue et al., 2020). Basal-like A phenotype was shown to be highly prevalent in metastatic advanced disease, enriched with squamous signatures and associated with increased genomic instability such as genome doubling and high *KRAS* imbalance (i.e., imbalance between wildtype and mutant *KRAS* alleles favoring the mutant *KRAS* allele) (Chan-Seng-Yue et al., 2020). However, if a relationship exists between high imbalance in *KRAS*, genomic doubling and increased expression of squamous signature is still unclear. Despite that, it suggests that increased mutant *KRAS* dosage may lead to increased KRAS signaling and promote metastasis.

Additional studies at single cell resolution revealed that most PDAC tumors harbor both classical and basal-like tumor cells, thereby showing that the two subtypes co-exist in a single tumor (Juiz et al., 2020). This demonstration of high intra-tumor heterogeneity even at the subtype level further highlights the need for personalized oncology. Treatment targeting for example basal-like tumor cells will not be sufficient if both subtypes are present in most tumors, demonstrating the complexity of targeting tumor cell subtypes.

In summary, distinct subtypes identified in massive sequencing studies revealed pronounced heterogeneity in PDAC with also less prevalent mutations, genome doubling, copy number changes, and chromosome alterations contributing to tumorigenesis besides mutations in the four major PDAC drivers (*KRAS, CDKN2A, SMAD4*, and *TP53*). This heterogeneity is the main reason for the current treatments not being more effective in PDAC, as the differences that prevail at molecular and cellular level of each tumor confer different susceptibility to treatment.

This demonstrates that while we need targeted therapies that specifically target the dysregulated molecular mechanisms driving tumor progression, there will never be a “one size fits all” solution in PDAC but a need for individualized treatment, for truly personal oncology.

## Combination Therapies for Undruggable KRAS

90% of PDAC possess mutations in the proto-oncogene *KRAS* (Almoguera et al., 1988; Raphael et al., 2017) and *KRAS* mutations which were shown to initiate the disease (Hingorani et al., 2005). In addition to *KRAS* mutation, *KRAS* amplification also contribute for disease progression in cancers including PDAC (Silverman et al., 1990; Liu et al., 1998; Heidenblad et al., 2002; O’Hagan et al., 2002; Aguirre et al., 2003; Yamada et al., 2008). Given the strong *KRAS* oncogene addiction of PDAC (Eser et al., 2014; Zeitouni et al., 2016), it appears as the obvious aim for targeted PDAC therapy.

KRAS is a small GTP binding protein, localized at the lipid rich cell membrane and cycling between GDP-bound inactive and GTP-bound active states. Activated KRAS triggers a signaling cascade by activating further downstream kinase effectors such as mitogen-activated protein 3 kinases (MAP3K) RAF, which then activate MAP2K kinases like MEK, activating MAP kinases (MAPK) like ERK, activating finally transcription factors that regulate gene expression changes involved in cell cycle regulation, tissue repair, angiogenesis, and differentiation (Ullrich and Schlessinger, 1990; Adjei, 2001; Lemmon and Schlessinger, 2010).

Researchers in the academic and pharmaceutical arena are trying for over four decades to discover a drug that effectively targets mutated RAS. A crucial amino acid is the glycine at position 12 which upon mutation is often replaced with another amino acid. The consequence is a prevention of GTP hydrolysis, constantly favoring the active GTP-bound state activating the downstream signaling cascade responsible for cell proliferation and survival (Scheffzek et al., 1997). Inhibitors have been developed that target mutant KRAS^G12C^ protein which were shown to be effective for lung cancer patients (Ostrem et al., 2013; Lito et al., 2016; Janes et al., 2018). However, in PDAC this mutation is only found in around 2% of cases, with *KRAS* ^*G12D*^ being the most common driver mutation (Cox et al., 2014). But despite tiring efforts, no clinically effective inhibitor for KRAS^G12D^ has been available for PDAC patients yet (Cox et al., 2014).

Efforts to develop effective KRAS inhibitors failed on one hand due to difficulties in targeting KRAS directly. Its high intrinsic affinity for GTP prevents the binding of competitive GTP inhibitors (Karnoub and Weinberg, 2008; Stephen et al., 2014). Another reason for the failure of KRAS inhibitors is that PDAC cells gain resistance based on other compensatory pathways (Karnoub and Weinberg, 2008; Stephen et al., 2014).

So indirect approaches to target KRAS appear to be required and one option would be targeting its downstream effector signaling. Several studies have provided compelling reason to consider the RAS-RAF-MEK-ERK pathway to target mutant KRAS-driven PDAC (Chen et al., 2016; Foster et al., 2016; Hayes et al., 2016; Riquelme et al., 2016; Raphael et al., 2017).

In a mutant KRAS-driven model of PDAC it was shown that mutant *KRAS* can be phenocopied by replacement with an activated mutant *BRAF* ^*V600E*^ allele (Collisson et al., 2012). In the rare cases of PDAC with wild type *KRAS*, a high percentage have a *BRAF* ^*V600E*^ mutation (Witkiewicz et al., 2015; Raphael et al., 2017; Seghers et al., 2020). However, BRAF selective inhibitors are effective only in BRAF mutant tumor models and have paradoxically activated ERK signaling in *KRAS* mutant or *RAS/RAF* wild type tumor models (Hatzivassiliou et al., 2010; Poulikakos et al., 2010). This supports the notion that these inhibitors might have opposing roles and function either as an inhibitor or activator of the same signaling pathway depending on the cellular context.

Inhibition of ERK as monotherapy is limited by normal cell toxicity and cancer cells often acquire resistance by ERK reactivation through loss of an ERK-dependent negative feedback mechanism (Ozkan-Dagliyan et al., 2020; Klomp et al., 2021). MEK inhibitors also caused feedback reactivation of ERK and have shown limited to no activity in *KRAS* mutant cancers (Samatar and Poulikakos, 2014).

Combination therapy inhibiting distinct nodes of the same pathway concurrently was suggested as a strategy to treat *KRAS* mutant PDAC (Ozkan-Dagliyan et al., 2020). Lower doses of RAF-ERK inhibitory combinations were shown to exhibit a synergistic suppression of activated ERK and caused cell cycle arrest and apoptosis (Ozkan-Dagliyan et al., 2020). Similarly, studies have also shown that combined RAF-MEK inhibition would overcome ERK reactivation in *KRAS* mutant or wild type cancer lines (Lamba et al., 2014; Yen et al., 2018), implicating this as a potential strategy for PDAC. Supporting studies from BRAF mutant melanoma have shown that combined BRAF-MEK inhibition is effective, exhibiting synergistic growth suppression and delaying onset of resistance, and has been clinically approved for this cancer type (Flaherty et al., 2012; Larkin et al., 2014; Long et al., 2014; Dummer et al., 2018). There is an ongoing clinical trial evaluating a pan-RAF inhibitor in combination with ERK or MEK inhibitors in *KRAS*-mutant non-small cell lung cancer and *NRAS*-mutant melanoma which will hopefully provide insights about RAF-ERK vs RAF-MEK inhibitory combinations (NCT02974725) (Ozkan-Dagliyan et al., 2020).

In summary, while mutant KRAS itself appears to be undruggable, identifying combination therapies that overcome pathway reactivation by targeting distinct compensatory mechanisms cancer cells use for their survival when KRAS signaling is suppressed is crucial for the development of effective combinatorial strategies that would result in synergistic tumor regression effects.

## PI3K Pathway Drives PDAC Progression and Compensates When KRAS Signaling Is Suppressed

Another signaling pathway activated by KRAS and implicated in PDAC is the PI3K/AKT pathway (Edling et al., 2010). Phosphoinositide 3 kinase (PI3K) describes a family of heterodimeric enzymes composed of a regulatory and a catalytic subunit. The catalytic subunits are classified into p110α, p110β, p110γ, and p110δ encoded by the genes *PIK3CA*, *PIK3CB*, *PIK3CG*, and *PIK3D* respectively (Hawkins et al., 2006). Upon activation, PI3K convert the cell membrane component phosphotidylinositol (4, 5) bisphosphate (PIP2) to phosphatidylinositol (3, 4, and 5) trisphosphate (PIP3) (Cantrell, 2001). PIP3 acts as an activating anchor for 3-phosphoinositide-dependent protein kinase 1 (PDK1) which in turn activates protein kinase B (PKB), also called AKT (Alessi et al., 1997; Currie et al., 1999). AKT activates further downstream signaling components such as the mechanistic target of rapamycin kinase (mTOR) (Cantrell, 2001; Janku, 2017). PI3K pathway activation plays a major role in cell cycle regulation, survival, and differentiation (Alessi et al., 1997; LoPiccolo et al., 2008). The tumor suppressor protein phosphatase tensin homolog (PTEN) converts PIP3 back to PIP2, acting as a negative regulator of the PI3K pathway and preventing cellular over proliferation (Maehama and Dixon, 1998).

PI3K was shown to be activated by oncogenic KRAS in both human and mouse models of PDAC (Jimeno et al., 2008; Kennedy et al., 2011; Ying et al., 2011). PDAC tumors have further been shown to have gain of function mutations in the oncogene *PIK3CA* or loss of function mutations in the tumor suppressor gene *PTEN* (Ying et al., 2011; Foo et al., 2013; Waddell et al., 2015). Oncogenic *PIK3CA* expression phenocopied KRAS driven PDAC progression (Payne et al., 2015). *PIK3CA* was also shown to regulate tumor immunogenicity and a genetic ablation of *PIK3CA* rendered mutant KRAS/TP53 driven pancreatic tumors more immunogenic by increasing the expression of major histocompatibility complex class 1 (MHC 1) and CD80, both of which are needed for T-cell stimulation (Sivaram et al., 2019). Almost 60–70% of PDAC is also associated with increased AKT activity, for example through *AKT2* oncogene amplification (Cheng et al., 1996; Ruggeri et al., 1998; Schlieman et al., 2003).

Oncogenic KRAS was also shown to drive PDAC progression through PDK1 (Eser et al., 2013). Inactivation of *PDK1* in epithelial cells of the pancreas significantly reduced tumor formation driven by KRAS^G12D^ (Eser et al., 2013). However, inactivation of *PDK1* using a recombinant strategy in epithelial cells of the lung have shown no decrease in progression of non-small cell lung cancer (NSCLC) (Eser et al., 2013). Further, in mutant KRAS driven NSCLC inhibition of PI3K-mTOR did not reduce tumor growth substantially (Engelman et al., 2008) but inhibition of cRAF, another effector of KRAS, decreased cancer progression (Blasco et al., 2011; Karreth et al., 2011). This demonstrates that each tissue has its own specific molecular events and signaling requirements for tumor progression. Hence, it is important to investigate the differences of the activated effector pathways driven by oncogenic KRAS in a tissue and context specific manner (Eser et al., 2013).

Distinct mutations are also associated with different biological potency of driving cancer progression, as well as conferring distinct therapeutic vulnerabilities. Commonly found PDAC mutations KRAS^G12D^ and KRAS^G12V^ were both shown to elevate macropinocytosis, a nutrient-scavenging metabolic activity critical for PDAC growth, through the key effector PI3Kα (p110α) (Hobbs et al., 2020). However, a rarer KRAS^G12R^ mutation, which is still found in about 20% of PDAC cases, does not interact with PI3Kα due to structural deformity, but the mutant cells still elevate macropinocytosis through a compensatory mechanism that activates the PI3K pathway through upregulation of another PI3K isoform, namely PI3Kγ (p110γ) (Hobbs et al., 2020). Hence, inhibitors selective to p110γ PI3K, but not p110α were effective in blocking macropinocytosis in KRAS^G12R^ driven PDAC cells, whereas for KRAS^G12D^ driven PDAC cells, both, a p110α or p110γ selective inhibitor, were effective. In addition, the drug sensitivity pattern to other targeted therapies differed between KRAS^G12R^ and KRAS^G12D^ mutant PDAC cells (Hobbs et al., 2020).

This study further illustrated that the distinct PI3K isoforms might have different roles in supporting cancer progression. Since these isoforms exist in both tumor and supporting stromal cells (Graupera et al., 2008; Thorpe et al., 2015; Conway et al., 2019), isoform specific targeting could enhance tumor regression and prevent off-target side effects in healthy tissues (Thorpe et al., 2015; Yap et al., 2015). In this line, combination therapies targeting EGFR and PI3Kα (p110α) in PDAC with high EGFR and AKT activity have shown promising efficacy (Wong et al., 2014).

Another study showed that p110α PI3K is required for KRAS-induced transformation of acinar to ductal metaplasia (ADM) via regulation of RAC1 (a known regulator of tumorigenesis) (Wu et al., 2014). Ablation of p110α but not p110β PI3K resulted in protection from tumorigenesis in a KRAS^G12D^ driven pancreatic tumor mouse model. However, ablation or long-term inhibition of p110α PI3K lead to an activation of downstream AKT, probably due to compensatory activity of other PI3K isoforms, suggesting that both isoform specific targeting and combinatorial therapies are important (Wu et al., 2014).

PDAC cells were shown to use the PI3K pathway as a compensatory mechanism for their survival. When *KRAS* was ablated, PI3K was shown to activate MAPK signaling and an unbiased chemical screen identified *KRAS* ablated cells sensitive to PI3K inhibition (Muzumdar et al., 2017). KRAS inhibition was shown to activate AKT through the mTORC2 complex (Brown et al., 2020). Combinatorial inhibition of KRAS^G12C^ and mTORC1/2 or MEK and mTROC1/2 however suppressed tumor growth in PDAC *in vitro* models and *in vivo* experiments (Brown et al., 2020), identifying another potential targeted combination to overcome resistance mechanisms.

In another study, a subset of mutant KRAS dependent PDAC cells acquired *de novo* resistance upon treatment with an ERK inhibitor through activation of the PI3K pathway, thereby overcoming ERK inhibition (Hayes et al., 2016). Modest anti-tumor activity was observed when MEK and PI3K were concurrently inhibited in mouse models of PDAC (Alagesan et al., 2015; Junttila et al., 2015). However, normal tissue toxicity is a limiting factor for combinatory inhibition of MEK and PI3K (Shimizu et al., 2012; Tolcher et al., 2015).

Multi-drug resistance pathways were shown to be activated by PI3K signaling in different cancers such as lung, breast, and chronic myeloid leukemia (Chen et al., 2018; Soltani et al., 2019; Wu et al., 2019). Chemoresistance through accelerated cell cycle processes was also credited to the activation of NF-κB, a downstream effector of the PI3K-AKT pathway, in several cancers including PDAC (Sui et al., 2014; Hu et al., 2015; Zhu et al., 2015; Eberle, 2019; Liu et al., 2020). Targeting the pathway component mTOR in cancer associated fibroblasts was demonstrated to reduce chemoresistance in PDAC (Duluc et al., 2015).

In summary, PI3K is another important signaling pathway involved in PDAC oncogenesis through various deregulations and a key player for the development of adaptive resistance to KRAS signaling inhibition, illustrating the need for combinatorial targeted treatment approaches beyond the KRAS-MAPK pathway.

## Metabolic Alterations Drive PDAC Progression

Several studies showed that PDAC cells tend to reprogram their metabolic activity to allow them to survive in the hypoxic tumor microenvironment (Commisso et al., 2013; Guillaumond et al., 2013; Son et al., 2013; Chini et al., 2014).

Increased glycolysis is a major hallmark acquired by cancer cells for their uncontrolled growth (Vander Heiden et al., 2009; Lu et al., 2012; Ying et al., 2012; Cai et al., 2013) and PDAC cells were shown to have an increased glycolysis conferred through the enzyme NADP(H) oxidase (NOX) (Lu et al., 2012). In a mutant KRAS driven mouse model of PDAC, the increase in glycolytic activity was driven by KRAS through the ERK-MAPK pathway and through loss of the oncogene *MYC* (Ying et al., 2012). Bryant et al., extended this observation to human PDAC in 2019 and showed that the glycolytic flux decreased upon both, KRAS suppression and ERK inhibition (Bryant et al., 2019).

Broad metabolic profiling stratified PDAC into different metabolic subtypes, lipogenic, and glycolytic, based on distinct metabolic reprogramming events and it was shown that each subtype has unique drug sensitivity profiles for specific classes of metabolic inhibitors (Daemen et al., 2015). The study also demonstrated that the lipogenic and glycolytic metabolic subtypes correlate with an epithelial and mesenchymal phenotype of PDAC respectively (Daemen et al., 2015).

The two major PDAC subtypes, classical and squamous, were shown to be driven by distinct metabolic phenotypes (Brunton et al., 2020). The worst prognostic squamous subtype of PDAC was shown to be highly catabolic, enriched with glycolytic transcripts and associated with increased glycolytic flux with high lactate secretion and decreased oxygen consumption (Bailey et al., 2016; Brunton et al., 2020). In homozygote *KRAS* ^*G12D/G12D*^ mutated lung cancer cells glucose metabolism was increased relative to *KRAS* ^*G12D/WT*^ heterozygous cells (Kerr et al., 2016). However, DNA sequencing analysis of PDAC established that *KRAS* ^*G12D*^ hetero- and homozygote cells were present across both PDAC subtypes (Brunton et al., 2020), therefore, another genetic or epigenetic event might have acted as a switch driving these metabolic changes in PDAC. It was shown that epigenetic loss of the genes *HNF4A* (hepatocyte nuclear factor 4 alpha) and *GATA6* (GATA binding protein 6), drives the metabolic reprogramming and switches the PDAC cells from classical to squamous subtype with increased glycolytic flux (Brunton et al., 2020).

Other metabolic pathways, namely macropinocytosis, an endocytic mechanism that PDAC cells utilize to accumulate essential amino acids (Commisso et al., 2013; Andreasson et al., 2020) and cholesterol metabolism (Chen et al., 2015; Guillaumond et al., 2015) also play significant roles in PDAC progression. Epidemiological and other studies have shown that statins, i.e., drugs reducing serum concentration of cholesterol, can be used to reduce the tumor load and improve the survival of PDAC patients (Walker et al., 2015; Wu et al., 2015; Huang et al., 2016). The signaling pathways that drive this dysregulation in metabolism and the order of direct and indirect signaling events is yet to be deciphered.

Another metabolic alteration associated with KRAS suppression or ERK inhibition in PDAC is impaired mitochondrial function (Viale et al., 2014; Kashatus et al., 2015; Bryant et al., 2019). A study performed in mouse PDAC cells showed that the PDAC cells resistant to oncogene ablation of *KRAS* ^*G12D*^ relied on increased mitochondrial respiration for survival (Viale et al., 2014). Mitochondrial fission, which includes fragmentation of mitochondrial matrix, was shown to be associated with KRAS induced transformation. Inhibition of the ERK-MAPK pathway resulted in increased mitochondrial fusion through blocking of mitochondrial fission, also reflecting increased oxygen consumption (Kashatus et al., 2015; Serasinghe et al., 2015). However, another study did not observe an increase in oxygen consumption with acute KRAS suppression or ERK inhibition in both human and mouse PDAC cell lines, instead the mitochondrial activity persisted at a lower level (Bryant et al., 2019). Potentially, ERK inhibition also suppressed the genes involved in mitochondrial biogenesis (Bryant et al., 2019).

Elevated autophagy was identified as a compensatory metabolic pathway that PDAC cells use when the glycolytic pathway and mitochondrial function were ablated through inhibition of KRAS signaling (Guo et al., 2011; Yang et al., 2011, 2014; Bryant et al., 2019; Kinsey et al., 2019). When KRAS was ablated by either siRNA, chemical inhibitors, or in a doxycycline-inducible model, all resulted in an elevated autophagic flux (Bryant et al., 2019). This metabolic alteration of increased autophagic flux was shown to be mediated mainly by the RAS-RAF-MEK-ERK pathway and the combination of ERK/MEK and autophagic inhibitors such as hydroxychloroquine resulted in a synergistic anti-tumor activity (Bryant et al., 2019; Kinsey et al., 2019). Several clinical trial studies have been initiated for this combined MEK/ERK and autophagy inhibition in RAS-mutant cancers including PDAC (NCT03825289, NCT04132505, NCT04214418, NCT04386057, and NCT04145297; https://clinicaltrials.gov).

In summary, metabolic alterations also drive PDAC progression and act as compensatory mechanisms under treatment. Therefore, metabolic dysregulation should also be considered in treatment strategies and there are indeed clinical studies underway that target autophagy in addition to KRAS signaling.

## Epigenetic Alterations Drive PDAC Progression

Cancer cells attain malignant transformation via genomic instability, which serves as one of the main hallmarks for disease progression (Yao and Wei, 2014). Genomic instability can occur at both, the genetic and epigenetic level (Putiri and Robertson, 2011) and it increases as the tumor progresses. Epigenetics refers to the control of gene expression without changing the DNA sequence, e.g., via chemical modifications like methylation of the DNA, that can be passed on in cell divisions (Jones and Baylin, 2007; Akhavan-Niaki and Samadani, 2013; Ciernikova et al., 2020; Alonso-Curbelo et al., 2021). Epigenetic changes like gene silencing by promoter hypermethylation or gene overexpression by promoter hypomethylation were shown to impact cancer progression (Jones and Baylin, 2007).

Epigenetic drugs were shown to reduce tumorigenicity in pre-clinical models and some are already used in clinical trials (Hessmann et al., 2017; Ciernikova et al., 2020; Morel et al., 2020). However, so far, they are associated with limited efficacy and not successful as monotherapy.

Epigenetic changes should also be considered for biomarker discovery. CpG island (i.e., genomic regions with many CpG dinucleotide repeats) methylation that leads to loss of gene expression is widely studied in cancer and known to be an efficient biomarker since the 1990’s (Jones and Baylin, 2002, 2007). Studies have also demonstrated that it is possible to detect DNA methylation markers in liquids secreted by the pancreas (“pancreatic juice”) of PDAC patients which can be used for PDAC diagnosis (Matsubayashi et al., 2006; Kisiel et al., 2015).

Several studies have shown that aberrant hyper- and hypomethylation of specific genes contribute to PDAC development and progression (Ueki et al., 2000, 2001; Sato et al., 2003a, 2005). Genome wide studies have identified 1,658 loci which were differentially methylated in PDAC when compared to normal pancreas (Vincent et al., 2011b). Another study using a microarray platform to profile DNA methylation in a genome wide manner showed that hundreds of promoters and CpG island were aberrantly methylated in PDAC cells (Omura et al., 2008). Utilizing the DNA-hypomethylating agent 5-Aza-dC and comparing the gene expression profiles before and after treatment in PDAC cell lines, resulted in the identification of several genes that showed abnormal methylation patterns at both CpG rich and CpG poor islands. This abnormal methylation was also detected in a selection of those genes in cancer tissue and pancreatic juice samples from PDAC patients (Sato et al., 2003b).

Genes with epigenetic dysregulation in PDAC include the cell cycle regulator *CDKN2A* and tumor suppressor *E-cadherin*, which were shown to be silenced by promoter DNA hypermethylation (Fukushima et al., 2002; Vincent et al., 2011b). Genes involved in the process of epithelial-mesenchymal transition (EMT), an important step in cancer progression, such as *TWIST1* and *BMP3* were found to be over-expressed with promoter hypomethylation (Tew et al., 2020).

An epigenetic mechanism behind an observed relationship between KRAS dependency and EMT in PDAC cell lines had also been suggested in an earlier study. The study found that KRAS dependent PDAC cells had a better differentiated epithelial phenotype with higher expression of the epithelial marker E-cadherin and upon EMT, KRAS dependency was reduced but there was no association with the mutational status of tumor suppressor and oncogenes other than KRAS, potentially suggesting an epigenetic mechanism behind the loss and gain of KRAS dependency (Singh et al., 2009).

KRAS also plays a role in gene expression regulation at the epigenetic level (Gazin et al., 2007; Serra et al., 2014). RAS was shown to mediate the epigenetic silencing of genes such as *FAS*, coding for the Fas cell surface death receptor which is needed for apoptosis in KRAS transformed mouse NIH3T3 and KRAS transformed human HEC1A cell lines (Gazin et al., 2007). A study performed in cell lines from lung cancer models showed that changes in DNA methylation were associated with mutant KRAS^G12V^ overexpression influencing the expression of genes encoding for factors mainly involved in the biological processes of differentiation and development (Tew et al., 2020). In the same study, KRAS mutant and dependent pancreatic cancer lines also exhibited over 8,000 differential methylations of CpGs upon *KRAS* knockdown and differentially methylated promoters also showed an enrichment of genes involved in differentiation and development (Tew et al., 2020). Interestingly, while this study demonstrated an effect of KRAS on epigenetic changes, they also found that the observed DNA methylation changes were mostly random and strongly influenced by cell type, contributing to high heterogeneity between cell lines (Tew et al., 2020). Another noteworthy finding was that the ERK pathway, which is the main effector signaling pathway for uncontrolled proliferation in mutant KRAS driven PDAC (Hayes et al., 2016), was not responsible for the associated methylation changes over the observed short time frame (Tew et al., 2020), leaving the responsible signaling pathway to be deciphered.

The differences between the PDAC subtypes such as classical and squamous involve changes in the epigenetic landscape (Bailey et al., 2016; Lomberk et al., 2018; Somerville et al., 2018). The worst prognostic basal/squamous subtype of PDAC was shown to be highly hypermethylated (Bailey et al., 2016; Miyabayashi et al., 2020). This subtype was also associated with mutations in genes of several epigenetic regulators such as *KDM6A*, *KMT2C,* and *KMT2D* (Collisson et al., 2011; Bailey et al., 2016; Andricovich et al., 2018). Loss of gene expression that drive endodermal lineage specification, namely *HNF4A* and *GATA6,* through epigenetic silencing with promoter hypermethylation were shown to drive the cancer to a more squamous-like subtype (Brunton et al., 2020). Several studies have also shown that the cancer progresses to an invasive basal/squamous subtype through epigenetic modifications at the level of super enhancers mediated by the transcription factor TP63 (Andricovich et al., 2018; Hamdan and Johnsen, 2018; Somerville et al., 2018). However, more studies are needed to determine whether there is a consistent change in methylation patterns in the subtypes of PDAC and whether oncogenic KRAS controls the epigenomic changes that are crucial for cancer phenotype.

Epigenetic regulation has been shown to impact drug response. One example is the promoter methylation of the gene *MGMT* (O-6-methylguanine-DNA methyltransferase), coding for a DNA repair enzyme, which was shown to increase sensitivity to drugs such as carmustine and temozolomide in gliomas (Esteller et al., 2000; Hegi et al., 2005). In a multi-omic analysis on PDAC xenografts, the cholesterol transporter NPC1L1 (NPC1 like intracellular cholesterol transporter 1) was identified as a potential therapeutic target and found to be highly epigenetically deregulated. High levels of NPC1L1 were observed in PDAC tumors of the classical subtype and low levels in the basal/squamous subtype. Interestingly, the NPC1L1 inhibitor ezetimibe was more effective on basal subtype PDAC cells than classical subtype, as the inhibitor also functions as a cholesterol competitor, thereby also supporting the implication for metabolic approaches in PDAC treatment (Nicolle et al., 2017).

A potential resource to identify additional epigenetic deregulated targets are databases. Pancreatic Cancer Methylation Database (PCMdb) (http://crdd.osdd.net/raghava/pcmdb/) is a database that was developed to support the identification of DNA methylation-based biomarkers in pancreatic cancer. It provides data on the DNA methylation status of 4,342 genes from PDAC cell lines and tissues (Nagpal et al., 2014). It further integrated drug resistance data from another database, the Cancer Drug Resistance Database (CancerDR) (http://crdd.osdd.net/raghava/cancerdr/) which provides information on 148 anti-cancer drugs and their pharmacological profiling across distinct cancer cell lines (Kumar et al., 2013). These different database tools will facilitate the concept of personalized medicine and highlight the importance of utilizing DNA methylation of genes as an effective predictor of response to chemotherapeutic cancer drugs (Nagpal et al., 2014).

In summary, epigenetic regulation has implications in the phenotype, drug response and clinical outcome of PDAC and epigenetic alterations can be considered as another major driving factor in PDAC oncogenesis. Therefore, targeting epigenetic changes in mutant KRAS dependent PDAC could be a new therapeutic intervention.

## Wnt Pathway Is Another Factor in PDAC Chemoresistance

The Wnt signaling pathway is crucial for stem cell maintenance, tissue repair, embryonic development and differentiation and plays a vital role in pancreatic organ development (Clevers et al., 2014). For pancreatic specification during embryonic development, Wnt pathway inhibition is needed, while activation of the Wnt pathway is required for the growth and maintenance of the organ and differentiation of pancreatic progenitor cells into exocrine and endocrine lineages (Murtaugh and Kopinke, 2008a; Murtaugh, 2008).

The Wnt pathway can be divided into either canonical or non-canonical pathways (Miao et al., 2013). In the canonical Wnt signaling pathway, the central molecule is β-catenin (Miao et al., 2013). Upon binding of a Wnt-protein ligand to Frizzled receptors in association with the co-receptor lipoprotein receptor–related protein (LRP)-5/6, several downstream signaling cascades occur resulting in the accumulation of β-catenin and its translocation to the nucleus where it binds with the transcription factor TCF and activates the transcription of Wnt pathway target genes (Nakamura et al., 2003; Sano et al., 2016).

Mutations of β-catenin are uncommon in PDAC (Bailey et al., 2016). However, *in vitro,* and *in vivo* studies have shown that the canonical Wnt signaling pathway influences PDAC tumorigenesis, and the majority of PDACs are characterized by an upregulated Wnt/β-catenin transcriptional signature (Zeng et al., 2006; Magliano et al., 2007).

The Wnt signaling pathway has been described to promote resistance to apoptosis and maintenance of cancer stem cells (CSCs), resulting in the pathogenesis of PDAC (Modi et al., 2016) and is also upregulated in the worst prognostic squamous subtype of PDAC (Fang et al., 2017).

It was shown that higher expression of canonical Wnt ligands such as *WNT2*, *WNT5A*, and *WNT7A* are highly implicated in pancreatic carcinogenesis (Jiang et al., 2014; Bo et al., 2016; Wu et al., 2018; Makena et al., 2019). Also, other Wnt pathway associated genes, such as Wnt antagonists *DKK1* (Dickkopf-1) and *HMGA2,* a member of the non-histone chromosomal high mobility group (HMG), played an important role in PDAC oncogenesis (Tang et al., 2018). In addition, activation of the non-canonical Wnt signaling pathway through GATA-binding factor 6 (GATA6), cyclin-dependent kinase 8 (CDK8) and R-spondin lead to a progression of PDAC (Zhong et al., 2011; Xu et al., 2015; Chartier et al., 2016). GATA6 promoted Wnt activation and PDAC growth through transcriptional downregulation of the secreted Wnt inhibitor DKK1 (Zhong et al., 2011).

Targeting the Wnt/β-catenin pathway is an actively prosecuted strategy in the treatment of PDAC (Krishnamurthy and Kurzrock, 2018). Several novel inhibitors of the Wnt/β-catenin pathway have been developed as well as monoclonal antibodies against Wnt ligands to block their oncogenic activity (Krishnamurthy and Kurzrock, 2018). The monoclonal antibody vantictumab (OMP-18R5), for instance, showed growth inhibition in breast, pancreatic, colon, and lung cancer xenograft models (Smith et al., 2013). Vantictumab is effective against PDAC in transgenic and xenograft models alone or synergistically with chemotherapy, including gemcitabine or nab-paclitaxel (Gurney et al., 2012).

Wnt antagonists have been applied successfully to sensitize also for other drug treatments, such a taxane, in PDAC models (Fischer et al., 2017; Makena et al., 2019). Treatment with taxanes, a class of chemotherapeutics that inhibits mitotic spindle degradation, as monotherapy had no significant effect on tumor cells with high Wnt signaling activity, leading to tumor cell accumulation (Fischer et al., 2017; Makena et al., 2019). However, sequential administration of Wnt antagonists such as vantictumab and ipafricept followed by taxane treatment prevented this selection for Wnt-active taxane-resistant tumor cells and demonstrated superior antitumor activity in PDAC models (Fischer et al., 2017). This combination strategy was also incorporated into PDAC phase I clinical trials (NCT02005315 and NCT02050178).

It was also shown that KRAS signaling increases the interaction of β-catenin with cAMP response element binding protein (CREB)-binding protein (CBP), interestingly creating a potential point for therapeutic intervention (Manegold et al., 2018). In this study, the specific small molecule CBP/β-catenin antagonist ICG-001 was used to investigate its effect on human PDAC cells in both an orthotopic mouse model and a human patient-derived xenograft (PDX) model of PDAC. ICG-001 sensitized PDAC cells and PDX tumors to gemcitabine treatment which significantly decreased the tumor volume (Manegold et al., 2018). ICG-001 has demonstrated anti-tumor effects in several tumor types (Grigson et al., 2015). Another inhibitor, PRI-724, was also shown to block the interaction between β-catenin and CBP (Lenz and Kahn, 2014). In PDAC cell lines, PRI-724 promoted differentiation of chemotherapy-resistant cancer stem cells and decreased the metastatic potential (Lenz and Kahn, 2014). A phase I clinical trial demonstrated that PRI-724 can be safely administered in combination with gemcitabine in PDAC patients (Ko et al., 2016).

The E3 ligase RNF43 (Ring Finger Protein 43) inhibits Wnt signaling by ubiquitinating Frizzled receptors for degradation (Tu et al., 2019). Mutations in *RNF43* occur in approximately 5–7% of PDAC (Aguilera and Dawson, 2021) and may serve as a useful biomarker for patient selection during clinical development of Wnt inhibitors, as it was shown that RNF43 mutant PDAC cell lines and xenograft models were sensitive to the porcupine inhibitor LGK974 (Jiang et al., 2013). Porcupine is an acyltransferase required for the secretion and activity of Wnt ligands (Proffitt and Virshup, 2012) and its inhibitor LGK974 is currently tested in a phase I clinical trial in several cancers including PDAC (NCT01351103).

The microRNA MiR-29a was reported to induce gemcitabine chemoresistance via the canonical Wnt signaling pathway (Nagano et al., 2013). The inhibition of Wnt signaling could reverse this chemoresistance to gemcitabine in PDAC (Nagano et al., 2013). MiR-33a, on the other hand, was reported to increase gemcitabine sensitivity in human PDAC cells by downregulating the nuclear translocation of β-catenin (Liang et al., 2015). The tyrosine kinase inhibitor masitinib also increased the sensitivity of pancreatic cell lines to gemcitabine by downregulation of the Wnt/β-catenin pathway (Jia and Xie, 2015). Wnt/β-catenin signaling is also associated with 5-FU resistance in PDAC cells as demonstrated in a 2018 study by Cao et al., which showed that the inhibition of glypican-4 (GPDAC4), a member of the glypican family and regulator of Wnt/β-catenin signaling, increased sensitivity to 5-FU in PDAC cells (Cao et al., 2018).

In summary, the Wnt signaling is another pathway involved in PDAC tumorigenesis and development of chemoresistance and targeting this pathway in combinatorial treatment approaches is an actively pursued strategy.

## Duality Role of TGF-β Pathway in PDAC

The transforming growth factor β (TGF-β) pathway plays an important, context-dependent role as both a tumor suppressor and a promoter of PDAC and is altered in 47% of PDAC cases (Bailey et al., 2016; Dardare et al., 2020). SMAD4 is an essential signal transducer of the canonical TGF-β pathway (Dardare et al., 2020) and is inactivated in approximately 60% of PDAC cases (Hahn et al., 1996). Thus, precision targeting of the TGF-β/SMAD4 pathway could be critical in the treatment of PDAC (Ahmed et al., 2019).

An alteration in *SMAD4* is generally associated with worse overall survival in both primary and metastatic PDAC demonstrating the importance of the canonical TGF-β pathway (Singh et al., 2011; Yamada et al., 2015; Shugang et al., 2016; Zhao et al., 2018). The squamous molecular subtype also presents activation of TGF-β signaling pathway (Bailey et al., 2016).

In normal pancreatic cells and in stages I and II of PDAC, TGF-β signaling acts as a tumor suppressor inhibiting cell proliferation (Glazer et al., 2014; Dardare et al., 2020). On the other hand, TGF-β signaling has been shown to have tumorigenic activity in many late-stage malignancies, including PDAC, due to severe dysregulations, suggesting an explanation for the apparent TGF-β paradox (Principe et al., 2014; Melzer et al., 2017).

Upregulated and overexpressed TGF-β has been shown to induce stromal proliferation in PDAC tumor microenvironment, promote EMT leading to metastases and consequently is a potential target for cancer therapy (Pickup et al., 2013; David et al., 2016; Hussain et al., 2018). TGF-β targeted therapy is established for various human cancers and data from several preclinical and clinical studies indicates that TGF-β blockade could be effective in the treatment of PDAC as well (Ahmed et al., 2019).

There are three possible approaches to target TGF-β signaling: inhibition at the translational level, inhibition at the ligand-receptor level, and inhibition of receptor-mediated signaling (Massagué, 2008). The goal of each of these targeted therapy approaches is to inhibit the tumor-promoting function and maintain the tumor-suppressive function of TGF-β (Haque and Morris, 2017). Another approach is to target TGF-β induced EMT that plays a critical role in PDAC progression and metastatic disease development (Alvarez et al., 2019).

The use of anti-TGF-β-based therapies in phase I and II clinical trials in metastatic PDAC highlights the importance of understanding the role of TGF-β in PDAC progression (Alvarez et al., 2019). A phase I/II clinical study has shown a survival benefit in PDAC and melanoma using AP-12009, an antisense oligonucleotide that acts directly against the mRNA of TGF-β2 (Nemunaitis et al., 2006; Oettle et al., 2011). Monoclonal antibodies targeting the ligand-receptor binding and preventing subsequent signaling of the TGF-β pathway (Gomez-Puerto et al., 2019) are under clinical investigation such as lerdelimumab (CAT-152, Trabio™ for TGF-β2) and metelimumab (CAT-192 for TGF-β1) (Ahmed et al., 2019).

Immunological pathways have proven to be successful targets in the treatment of other cancers, but not in PDAC (Hilmi et al., 2018). It was previously shown that TGF-β exerts an immunosuppressive function in the tumor immune microenvironment by antagonizing interleukin 15-mediated proliferation of natural killer (NK) cells (Wilson et al., 2011). This immunosuppressive function of NK and T cells through the SMAD-dependent canonical TGF-β pathway is another key role for TGF-β in promoting tumor immune evasion (Thomas and Massagué, 2005; Trotta et al., 2008). TGF-β signaling inhibition was shown to reverse this immune evasion function by restoring immune activity against tumor cells (Wilson et al., 2011).

Checkpoint inhibitors are another immune-targeting approach in treating cancer (Darvin et al., 2018). Monotherapy with checkpoint inhibitors failed to elicit efficacy in PDAC patients (Henriksen et al., 2019). However, there is growing evidence that combining checkpoint inhibitors with TGF-β signaling inhibition may prolong survival in several cancers (Bai et al., 2019).

The SMAD-independent or non-canonical TGF-β pathways include several branches that lead to activation of the Rho-like GTPase signaling pathway, PI3K/AKT pathway and/or MAP kinase pathway (Zhang, 2009). The MAP kinase pathway component ERK upregulates the cyclin-dependent kinase inhibitor 1A (CDKN1A), also called p21, thereby facilitating TGF-β-mediated cell cycle arrest (Tang et al., 2002; Torii et al., 2006). An ERK-induced cell cycle arrest through TGF-β/SMAD4 mediated *CDKN1A* upregulation was also observed in benign pancreatic cell lines (Principe et al., 2017). While ERK can contribute to tumor-suppressive TGF-β signaling in normal pancreatic epithelial cells, TGF-β-induced activation of ERK can be very damaging in the disease state (Principe et al., 2017). For this reason, the crossover between TGF-β and ERK signaling pathways deserves further attention, particularly regarding the functional switch from tumor-suppressive to tumor-promoting TGF-β signaling.

Therapies targeting TGF-β signaling have been investigated in the preclinical and clinical setting and have shown efficacy in PDAC (Shen et al., 2017). However, the paradoxical effects of TGF-β in human cancers are poorly understood, and although therapies targeting the TGF-β pathway have merit, it is important to ensure that they target only the tumor-promoting effect.

## Combined Targeted Phenotypic Approach Utilizing Patient Derived Organoids—A Promising Strategy for PDAC Treatment Development

So how can we translate our knowledge about the manifold dysregulations and compensatory mechanisms driving PDAC into more effective treatment options for patients?

**FIGURE 1:**
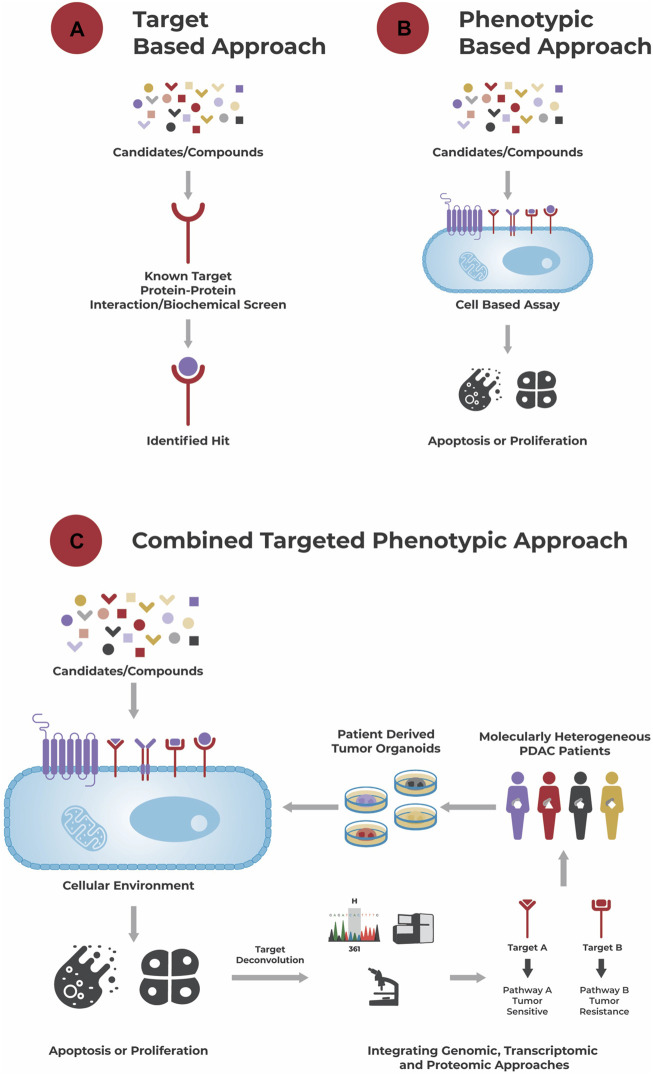
Different approaches to drug discovery. **(A)**: A target-based approach aims to identify a hit to a known target using a biochemical screen. **(B)**: A phenotypic-based approach identifies hits based on their effect in a cell-based assay, e.g., their ability to induce apoptosis or growth arrest, without necessarily knowing the compound’s biochemical target and its role in disease biology. **(C)**: A combined targeted phenotypic approach leverages both, a target-based and phenotypic approach. The effect of compounds is tested in a cellular environment and hits selected based on their effect on the cells’ phenotype. A powerful model system here are patient-derived organoids that recapitulate the genetic and molecular alterations of a patient’s tumor, and which are for example used in the approach of Reversed Clinical Engineering^®^. Then, using target deconvolution strategies like the integration of genomic, transcriptomic, and proteomic data, mechanisms (induced by the “hits”) conferring to drug sensitivity and/or resistance and suitable compounds for treatment can be identified for each individual patient tumor.

To identify effective treatments for cancer, knowledge about commonly found aberrations and dysregulations is invaluable. This “target-based approach” (Figure 1A) aims to identify compounds that specifically act on a previously defined target in the context of such known, often genetic mutation-based dysregulations to attack the tumor. Unfortunately, in PDAC targeting major genomic dysregulations such as those involved in KRAS or PI3K signaling did not result in effective therapies until now (Van Cutsem et al., 2004; Javle et al., 2010; Milroy and Ottmann, 2014).

An alternative “phenotypic based approach” (Figure 1B) aims to identify compounds that elicit a growth inhibitory or apoptotic effect on tumor cells in a cell-based assay without necessarily requiring knowledge about the tumor’s dysregulations and/or compounds’ mechanism of action. This functional approach takes the tumor’s complexity, interplay between different dysregulations, compensatory mechanisms and tumor heterogeneity into account and allows identification of compounds acting through unprecedented drug mechanisms (Moffat et al., 2017; Swinney and Lee, 2020).

One step further goes the “combined targeted phenotypic approach” (Figure 1C). After testing different single agents and combinatorial compounds in a phenotypic assay, allowing the rapid identification of responders and non-responders at a cellular level, integration of e.g., genomic, transcriptomic and/or proteomic data to those responders and non-responders allows identification of underlying drug sensitivity and/or resistance mechanisms (targets). Besides identification of effective compounds, potentially in a personalized manner, this approach can also be used for identification of biomarkers related to sensitivity or resistance towards a specific compound.

An essential prerequisite for the combined targeted phenotypic based approach is a suitable *in vitro* cancer model. Here patient-derived organoids are of specific interest and allow to identify the therapeutic responses of individual patient tumors including PDAC (Tiriac et al., 2018; Driehuis et al., 2019).

Patient-derived organoids are 3D cell culture models derived from small pieces of tissue, for example from a patient’s primary tumor or a metastasis (Silvestri et al., 2017; Schumacher et al., 2019; Pfohl et al., 2021). Culture conditions allow cancer stem-like cells to self-organize and form miniature versions of a patient’s tumor, i.e., organoids, that recapitulate the genetic, histologic, and molecular alterations of the tumor (Boj et al., 2015; Schütte et al., 2017; Driehuis et al., 2019), including its intra-tumor heterogeneity and functional phenotype (Schumacher et al., 2019; Pfohl et al., 2021). Further, patient-derived organoids were shown to be a suitable tool to predict treatment response in cancer patients (Vlachogiannis et al., 2018; Wensink et al., 2021) and can be used for high-throughput drug screens (Boehnke et al., 2016) and genetic manipulation (Boj et al., 2015).

Several studies have described the successful establishment of tumor organoid cultures from PDAC patients (Boj et al., 2015; Tiriac et al., 2018; Driehuis et al., 2019; Tuveson and Clevers, 2019). Organoid cultures were shown to recapitulate the hybrid nature of PDAC exhibiting distinct subtypes in a single tumor (Chan-Seng-Yue et al., 2020; Hayashi et al., 2020; Juiz et al., 2020).

Miyabayashi et al. performed an experiment in mice in 2020 where they carefully injected *in vitro* generated organoids directly into the pancreatic duct, the site where the pre-invasive pancreatic neoplasms originate and progress, and followed the patterns of intraepithelial neoplasms to gain cellular and molecular insights into the mechanisms promoting progression of PDAC (Miyabayashi et al., 2020). The organoids implanted in the duct, referred to as intraductally grafted organoid (IGO), gave rise to two different classes of neoplasm with distinct phenotypes, fast growing and slow growing, recapitulating the histologic heterogeneity of the tissue from which the organoids were derived. Fast progressor organoid-derived neoplasm recapitulated the basal-like or squamous subtype, showing invasiveness, migration from the duct, hyperactivation of the RAS pathway and activating cancer associated fibroblasts (CAFs). While the slow growing organoid-derived neoplasm recapitulated the classical or progenitor subtype and were more contained within the ducts (Miyabayashi et al., 2020). The study further showed that *KRAS* copy number was increased and the KRAS pathway was hyperactivated in the fast-growing organoids, whereas slow growing organoids showed increased expression of *GATA6*, a marker of the classical subtype, and low copy number for *KRAS* (Miyabayashi et al., 2020).

PDAC derived organoids were successfully used to assess drug sensitivity (Huang et al., 2015; Tiriac et al., 2018; Driehuis et al., 2019). Importantly, when treated with standard-of-care chemotherapeutics, the treatment response of PDAC-derived organoids was shown to correspond to that of the patients the models were originating from (Tiriac et al., 2018). This further supports the notion that patient-derived organoids have the potential to be used as personalized model of PDAC, predict therapy responses, thereby enabling prospective treatment selection and identification of new therapeutic strategies and can also be exploited for genomic and functional studies (Tiriac et al., 2018; Driehuis et al., 2019).

To allow utilization of the potential of patient-derived organoids as preclinical models but also to address the need for personalized oncology approaches, large collections of patient-derived tumor organoids and matching healthy (normal) organoids, i.e., organoid biobanks from various tumor entities including PDAC, were generated (van de Wetering et al., 2015; Drost and Clevers, 2018; Sachs et al., 2018; Calandrini et al., 2020; Botti et al., 2021). These biobanks contain genetically diverse cultures and are suitable to represent disease heterogeneity. Together with the integration of genomic and drug screening data, these organoid biobanks facilitate drug development by predicting the drug response and toxicity profiles for individual patients (van de Wetering et al., 2015), thereby including personalized medicine in the treatment discovery process. Furthermore, biobanks allow future assessment of newly developed drugs or combinations on the same tumor models and direct comparison with current treatment effects.

However, there are also limitations such as the need for optimization of culture methods for individualized normal and tumor cultures (Kondo et al., 2011). Organoid models can only be established under specific culture conditions, and these dependencies often reflect differences in the tumor’s mutational background (Fujii et al., 2016). However, in most cases the genetic background is not yet determined when establishing organoids from fresh patient tissues. This could lead to an underrepresentation of some alterations inhibitory to organoid derivation in the resulting biobanks. Poorly and moderately differentiated PDAC derived organoids could not be established in a biobank effort, further supporting the fact that distinct tumor subtypes require distinct culture conditions (Huang et al., 2015; Fujii et al., 2016).

Small scale drug screens on organoid biobanks already yielded promising results (Gao et al., 2014; van de Wetering et al., 2015; Sachs et al., 2018)) and further efforts to generate large, standardized, globally accessible banks of organoid models for the research community will continue to facilitate drug development and enhance personalized medicine.

Reverse Clinical Engineering^®^ is an approach that combines a phenotypic based assay with a target deconvolution strategy in a single system utilizing patient-derived organoids (Figure 1C). It is a technique that establishes patient-derived organoids from individual patient tumors, exposes them to a wide range of therapeutic regimen and integrates the treatment response with genomic, transcriptomic, and proteomic data. This approach does not only allow identification of functionally effective compounds for the individual tumor, meaning patient, but also deciphers the tumor’s molecular characteristics driving its therapy sensitivity or resistance. Reverse Clinical Engineering^®^ can be performed on an individual patient-derived organoid model, identifying the Achilles heel of this specific tumor, or on a collection of patient-derived organoids from a biobank of e.g., a specific cancer type (PDAC or other) to identify for example a new treatment strategy and/or biomarker for this tumor type. Either way, this approach of functionally profiling tumors in a potentially high through-put manner is of high potential for real personalized oncology (Pfohl et al., 2021) and a promising strategy to finally identify effective treatment options for PDAC patients.

Patient-derived organoids have the potential to connect compound screening and clinical trials. However, establishing distinct culture and assay conditions for each distinct tumor entity and constant supply of patient material for large screens remain a major challenge in using this combined targeted phenotypic approach with patient-derived organoids (Boehnke et al., 2016). Efficient establishment of organoid cultures for different entities, drug assays including initial seeding material and density, treatment regimen, assay reproducibility, evaluation of drug response (e.g., IC50, area-under-the curve, Z-score IC50), drug validation, and assay scalability remain technical constraints and all need to be optimized (Boehnke et al., 2016; Phan et al., 2019; Bergdorf et al., 2020) In addition, potential off-target toxicity cannot be assessed by organoid monocultures (Pfohl et al., 2021). Nevertheless, patient derived organoids are a powerful tool which can be further expanded to take the tumor microenvironment into account, known to impact drug response, through suitable co-culture systems with e.g., cancer associated fibroblasts or immune cells (Tsai et al., 2018).

## Discussion

PDAC is a highly heterogeneous disease with a complex combination of genetic, epigenetic, metabolic, and microenvironment dysregulations. Each patient exhibits distinct molecular alterations, different gene expression profiles and specific therapeutic responses.

Although genomic studies support the notion that PDAC is associated with only 4 driver mutations (*KRAS*, *CDKN2A*, *TP53*, and *SMAD4*) and perceived to be uniformly aggressive, a high level of clinical heterogeneity exists. High level of intrinsic cell plasticity, a random nature of genomic instability, constellations of genomic aberrations rather than a single event, dynamic epigenetic modulations and involvement and contribution of non-genetic features such as tumor-microenvironment with high desmoplastic stroma and low vascularity together contribute for the emergence of distinct phenotypic states in PDAC, making it highly heterogenous and exhibiting different treatment responses, often with pronounced drug resistance (Adams et al., 2019; Chan-Seng-Yue et al., 2020; Miyabayashi et al., 2020). Further, studies have shown that for most PDAC cases there is no association between genetic mutations and therapeutic responses (Witkiewicz et al., 2016; Knudsen and Witkiewicz, 2017), supporting the notion that multiple mechanisms of dysregulation need to be taken into account.

**FIGURE 2:**
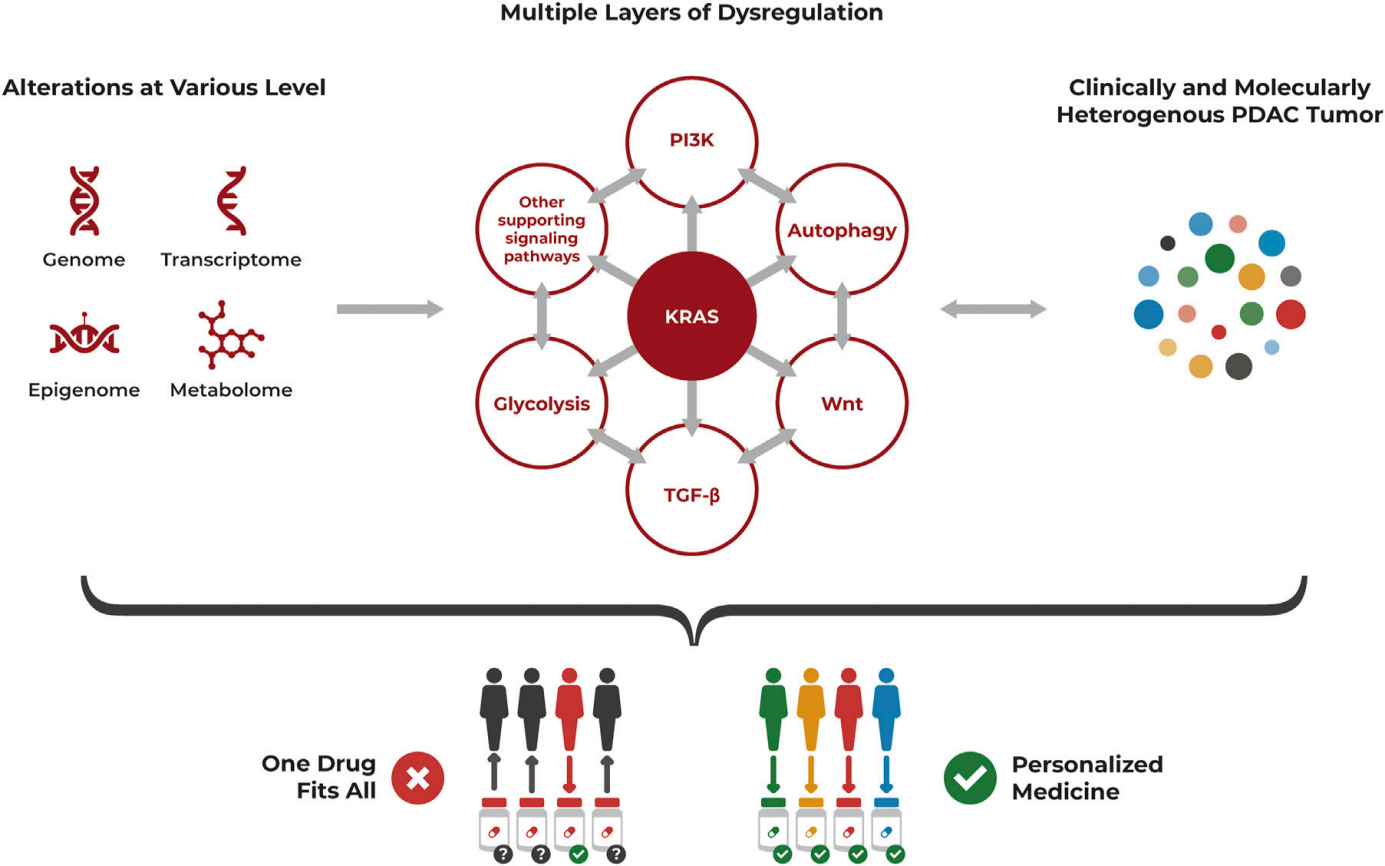
PDAC heterogeneity requires a personalized approach for treatment. PDAC is a highly heterogenous cancer at the cellular level, often driven by *KRAS* mutations but with multiple layers of dysregulation affecting each other, that also need to be considered. Alterations occur at various levels including genomic, transcriptomic, epigenomic and metabolomic aberrations. The resulting clinically and molecularly heterogenous tumor is unlikely to be successfully treated with a “one drug fits all” approach but requires a personalized approach specifically targeting its individual dysregulations.

The molecular und clinical heterogeneity of PDAC with distinct subtypes having different biologic and prognostic relevance, the alterations at various levels and multiple layers of dysregulation highlight the need for precision oncology to treat this complex cancer (Figure 2).

The pronounced heterogeneity of PDAC illustrates clearly that context matters and needs to be considered to develop effective treatment strategies. While 90% of PDAC contain a *KRAS* mutation (Almoguera et al., 1988; Raphael et al., 2017), strategies to target (just) this aberration did not yet lead to an improved PDAC treatment. As highlighted throughout this review, additional layers of dysregulation are present beyond genomic aberrations and other signaling pathways are deregulated in addition to KRAS, although often conferring with it. These dysregulations are not only cancer drivers by themselves but can also act in a compensatory way if one aberration is targeted by therapy.

Despite several inhibitors for distinct effector pathways developed and tested in pre-clinical models and clinical trials, adaptive resistance is still a major hurdle. When KRAS or MEK or mTORC1/2 are inhibited individually in PDAC cells, tumor cell plasticity and rapid adaptation to stress activates compensatory pathways and favors the survival of tumor cells (Brown et al., 2020). Hence, identifying co-vulnerabilities in these tumors often driven by mutant KRAS and developing combinatorial and concurrent inhibitory strategies will prevent the ability of PDAC to survive through compensatory growth promoting pathways. However, limitations in combinatorial approaches include the selection of appropriate doses of individual therapies for optimal efficacy, considering for the presence of off-target, normal cell toxicity and different molecular subtypes of PDAC exhibiting different sensitivity profiles. In addition, the mechanisms of how cancer cells get adapted to several layers of inhibition is a complex phenomenon which is yet to be understood.

Each PDAC tumor is unique. Even if alterations occurred on the same level or by the same mechanism, the exact changes within the cancer cells are still heterogenous (Tew et al., 2020). Further, the combinations of dysregulations and alterations are heterogenous. One of the main reasons for multi-drug resistance is the genetic and molecular heterogeneity of cancer cells (Gottesman et al., 2002). The impact of genetic alterations depends on oncogenic contexts and several studies showed that dysregulations should be analyzed in a tissue- or cancer entity specific manner (Eser et al., 2013; Foggetti et al., 2021). Therefore, to identify much needed, effective treatment strategies for PDAC, its heterogeneity needs to be considered. This translates into the need for personalized oncology with better patient stratification and treating individual patients separately by functionally profiling the individual tumors.

As a potential solution, this review highlighted the combined targeted phenotypic approach as a promising strategy to identify effective compounds using a cell-based assay, allowing to take this tumor heterogeneity and the context of various unique alterations into account—given suitable cellular models are used. A powerful tool for this approach are patient-derived organoids as these are suitable models for tumor heterogeneity and personalized oncology, but also for high-throughput drug screens (Pfohl et al., 2021).

In summary, the tumor driving mechanisms and associated molecular alterations are different for each tumor and due to this heterogeneity, it is basically impossible to develop a drug or treatment regimen that will be effective for all PDAC patients. Overcoming chemoresistance and identifying chemo sensitive signatures using pharmacogenomic profiling is emerging as a new way forward and reason for hope. Although target-based screening strategies that screened vast compounds for a single target have identified many “best in class” compounds, most of the “first in class” compounds in earlier decades were identified using phenotypic screening i.e., screening vast compounds on cells without a known target (Moffat et al., 2017). The sweet spot for the future lies in combining these target-based and phenotypic approaches in a single system.

Reverse Clinical Engineering^®^, a combined targeted phenotypic approach using patient-derived organoids, enables to identify effective drugs or drug combinations for an individual tumor within a cellular context, and deconvoluting the treatment’s mechanism of action by utilizing technological advances in genomics, transcriptomic and/or proteomics. The application of patient-derived organoids allows to incorporate tumor heterogeneity—a factor not yet sufficiently considered in PDAC therapy.

This approach of functionally profiling individual tumors in a potentially high throughput fashion utilizing automated liquid robotic system offers several advantages and has potential applications also in the rare disease space (Puca et al., 2018; Phan et al., 2019). It offers the ability to model also rare tumors and poses an opportunity to identify drugs in a disease and mechanism agnostic manner (Phan et al., 2019).

In conclusion, PDAC is a complex and heterogenous cancer with currently insufficient treatment options. Drug screening using patient-derived organoids of PDAC can factor in tumor heterogeneity and the context of the multi-layered dysregulations to identify new treatment strategies and transform PDAC therapy to personalized oncology.

## References

[B1] Adams C. R. Htwe H. H. Marsh T. Wang A. L. Montoya M. L. Subbaraj L. (2019). Transcriptional Control of Subtype Switching Ensures Adaptation and Growth of Pancreatic Cancer. Elife 8, e45313. 10.7554/elife.45313 31134896 PMC6538376

[B2] Adjei A. A. (2001). Blocking Oncogenic Ras Signaling for Cancer Therapy. JNCI J. Natl. Cancer Inst. 93, 1062–1074. 10.1093/jnci/93.14.1062 11459867

[B3] Aguilera K. Y. Dawson D. W. (2021). WNT Ligand Dependencies in Pancreatic Cancer. Front. Cel Dev. Biol. 9, 671022. 10.3389/fcell.2021.671022 PMC8113755 33996827

[B4] Aguirre A. J. Bardeesy N. Sinha M. Lopez L. Tuveson D. A. Horner J. (2003). Activated Kras and Ink4a/Arf Deficiency Cooperate to Produce Metastatic Pancreatic Ductal Adenocarcinoma. Genes Develop. 17, 3112–3126. 10.1101/gad.1158703 14681207 PMC305262

[B5] Ahmed S. Schwartz C. Dewan M. Xu R. (2019). The Promising Role of TGF- β/SMAD4 in Pancreatic Cancer: The Future Targeted Therapy. J. Cancer Treat. Diagn. 3, 1–7. 10.29245/2578-2967/2019/2.1141

[B6] Akhavan-Niaki H. Samadani A. A. (2013). DNA Methylation and Cancer Development: Molecular Mechanism. Cell Biochem Biophys 67, 501–513. 10.1007/s12013-013-9555-2 23508887

[B7] Alagesan B. Contino G. Guimaraes A. R. Corcoran R. B. Deshpande V. Wojtkiewicz G. R. (2015). Combined MEK and PI3K Inhibition in a Mouse Model of Pancreatic Cancer. Clin. Cancer Res. 21, 396–404. 10.1158/1078-0432.ccr-14-1591 25348516 PMC4447091

[B8] Alessi D. R. James S. R. Downes C. P. Holmes A. B. Gaffney P. R. J. Reese C. B. (1997). Characterization of a 3-phosphoinositide-dependent Protein Kinase Which Phosphorylates and Activates Protein Kinase Bα. Curr. Biol. 7, 261–269. 10.1016/s0960-9822(06)00122-9 9094314

[B9] Almoguera C. Shibata D. Forrester K. Martin J. Arnheim N. Perucho M. (1988). Most Human Carcinomas of the Exocrine Pancreas Contain Mutant C-K-Ras Genes. Cell 53, 549–554. 10.1016/0092-8674(88)90571-5 2453289

[B10] Alonso-Curbelo D. Ho Y.-J. Burdziak C. Maag J. L. V. Morris J. P. Chandwani R. (2021). A Gene-Environment-Induced Epigenetic Program Initiates Tumorigenesis. Nature 590, 642–648. 10.1038/s41586-020-03147-x 33536616 PMC8482641

[B11] Alvarez M. A. Freitas J. P. Mazher Hussain S. Glazer E. S. (2019). TGF-β Inhibitors in Metastatic Pancreatic Ductal Adenocarcinoma. J. Gastrointest. Canc 50, 207–213. 10.1007/s12029-018-00195-5 30891677

[B12] Amado R. G. Wolf M. Peeters M. Van Cutsem E. Siena S. Freeman D. J. (2008). Wild-TypeKRASIs Required for Panitumumab Efficacy in Patients with Metastatic Colorectal Cancer. Jco 26, 1626–1634. 10.1200/jco.2007.14.7116 18316791

[B13] Andreasson C. Ansari D. Ekbom F. Andersson R. (2020). Macropinocytosis: the Achilles' Heel of Pancreatic Cancer. Scand. J. Gastroenterol. 56, 177–179. 10.1080/00365521.2020.1855471 33280476

[B14] Andricovich J. Perkail S. Kai Y. Casasanta N. Peng W. Tzatsos A. (2018). Loss of KDM6A Activates Super-enhancers to Induce Gender-specific Squamous-like Pancreatic Cancer and Confers Sensitivity to BET Inhibitors. Cancer Cell 33, 512–526. e8. 10.1016/j.ccell.2018.02.003 29533787 PMC5854186

[B15] Bai X. Yi M. Jiao Y. Chu Q. Wu K. (2019). Blocking TGF-β Signaling to Enhance the Efficacy of Immune Checkpoint Inhibitor. Ott Vol. 12, 9527–9538. 10.2147/ott.s224013 PMC6857659 31807028

[B16] Bailey P. Chang D. K. Nones K. Johns A. L. Patch A. M. Gingras M. C. (2016). Genomic Analyses Identify Molecular Subtypes of Pancreatic Cancer. Nature 531, 47–52. 10.1038/nature16965 26909576

[B17] Bergdorf K. Phifer C. Bharti V. Westover D. Bauer J. Vilgelm A. (2020). High-throughput Drug Screening of fine-needle Aspiration-Derived Cancer Organoids. STAR Protoc. 1, 100212. 10.1016/j.xpro.2020.100212 33377106 PMC7757655

[B18] Bian B. Bigonnet M. Gayet O. Loncle C. Maignan A. Gilabert M. (2017). Gene Expression Profiling of Patient‐derived Pancreatic Cancer Xenografts Predicts Sensitivity to the BET Bromodomain Inhibitor JQ 1: Implications for Individualized Medicine Efforts. Embo Mol. Med. 9, 482–497. 10.15252/emmm.201606975 28275007 PMC5376755

[B19] Blasco R. B. Francoz S. Santamaría D. Cañamero M. Dubus P. Charron J. (2011). c-Raf, but Not B-Raf, Is Essential for Development of K-Ras Oncogene-Driven Non-small Cell Lung Carcinoma. Cancer Cell 19, 652–663. 10.1016/j.ccr.2011.04.002 21514245 PMC4854330

[B20] Bo H. Gao L. Chen Y. Zhang J. Zhu M. (2016). Upregulation of the Expression of Wnt5a Promotes the Proliferation of Pancreatic Cancer Cells *In Vitro* and in a Nude Mouse Model. Mol. Med. Rep. 13, 1163–1171. 10.3892/mmr.2015.4642 26648282 PMC4732830

[B21] Boeck S. Jung A. Laubender R. P. Neumann J. Egg R. Goritschan C. (2013). KRAS Mutation Status Is Not Predictive for Objective Response to Anti-EGFR Treatment with Erlotinib in Patients with Advanced Pancreatic Cancer. J. Gastroenterol. 48, 544–548. 10.1007/s00535-013-0767-4 23435671

[B22] Boehnke K. Iversen P. W. Schumacher D. Lallena M. J. Haro R. Amat J. (2016). Assay Establishment and Validation of a High-Throughput Screening Platform for Three-Dimensional Patient-Derived Colon Cancer Organoid Cultures. J. Biomol. Screen. 21, 931–941. 10.1177/1087057116650965 27233291 PMC5030729

[B23] Boj S. F. Hwang C.-I. Baker L. A. Chio I. I. C. Engle D. D. Corbo V. (2015). Organoid Models of Human and Mouse Ductal Pancreatic Cancer. Cell 160, 324–338. 10.1016/j.cell.2014.12.021 25557080 PMC4334572

[B24] Botti G. Bonito M. D. Cantile M. (2021). Organoid Biobanks as a New Tool for Pre-clinical Validation of Candidate Drug Efficacy and Safety. Int. J. Physiol. Pathophysiol Pharmacol 13, 17–21. 33815668 PMC8012858

[B25] Brown W. S. McDonald P. C. Nemirovsky O. Awrey S. Chafe S. C. Schaeffer D. F. (2020). Overcoming Adaptive Resistance to KRAS and MEK Inhibitors by Co-targeting mTORC1/2 Complexes in Pancreatic Cancer. Cel Rep. Med. 1, 100131. 10.1016/j.xcrm.2020.100131 PMC7691443 33294856

[B26] Brunton H. Caligiuri G. Cunningham R. Upstill-Goddard R. Bailey U. M. Garner I. M. (2020). HNF4A and GATA6 Loss Reveals Therapeutically Actionable Subtypes in Pancreatic Cancer. Cell Rep 31, 107625. 10.1016/j.celrep.2020.107625 32402285 PMC9511995

[B27] Bryant K. L. Stalnecker C. A. Zeitouni D. Klomp J. E. Peng S. Tikunov A. P. (2019). Combination of ERK and Autophagy Inhibition as a Treatment Approach for Pancreatic Cancer. Nat. Med. 25, 628–640. 10.1038/s41591-019-0368-8 30833752 PMC6484853

[B28] Cai Q. Lin T. Kamarajugadda S. Lu J. (2013). Regulation of Glycolysis and the Warburg Effect by Estrogen-Related Receptors. Oncogene 32, 2079–2086. 10.1038/onc.2012.221 22665055 PMC3435484

[B29] Calandrini C. Schutgens F. Oka R. Margaritis T. Candelli T. Mathijsen L. (2020). An Organoid Biobank for Childhood Kidney Cancers that Captures Disease and Tissue Heterogeneity. Nat. Commun. 11, 1310. 10.1038/s41467-020-15155-6 32161258 PMC7066173

[B30] Cantrell D. A. (2001). Phosphoinositide 3-kinase Signalling Pathways. J. Cel Sci 114, 1439–1445. 10.1242/jcs.114.8.1439 11282020

[B31] Cao J. Ma J. Sun L. Li J. Qin T. Zhou C. (2018). Targeting Glypican‐4 Overcomes 5‐FU Resistance and Attenuates Stem Cell-like Properties via Suppression of Wnt/β‐catenin Pathway in Pancreatic Cancer Cells. J. Cel Biochem 119, 9498–9512. 10.1002/jcb.27266 30010221

[B32] Chan-Seng-Yue M. Kim J. C. Wilson G. W. Ng K. Figueroa E. F. O’Kane G. M. (2020). Transcription Phenotypes of Pancreatic Cancer Are Driven by Genomic Events during Tumor Evolution. Nat. Genet. 52, 231–240. 10.1038/s41588-019-0566-9 31932696

[B33] Chartier C. Raval J. Axelrod F. Bond C. Cain J. Dee-Hoskins C. (2016). Therapeutic Targeting of Tumor-Derived R-Spondin Attenuates β-Catenin Signaling and Tumorigenesis in Multiple Cancer Types. Cancer Res. 76, 713–723. 10.1158/0008-5472.can-15-0561 26719531

[B34] Chen H. Qin S. Wang M. Zhang T. Zhang S. (2015). Association between Cholesterol Intake and Pancreatic Cancer Risk: Evidence from a Meta-Analysis. Sci. Rep. 5, 8243. 10.1038/srep08243 25649888 PMC4316166

[B35] Chen S.-H. Zhang Y. Van Horn R. D. Yin T. Buchanan S. Yadav V. (2016). Oncogenic BRAF Deletions that Function as Homodimers and Are Sensitive to Inhibition by RAF Dimer Inhibitor LY3009120. Cancer Discov. 6, 300–315. 10.1158/2159-8290.cd-15-0896 26732095

[B36] Chen Y. Wang T. Du J. Li Y. Wang X. Zhou Y. (2018). The Critical Role of PTEN/PI3K/AKT Signaling Pathway in Shikonin-Induced Apoptosis and Proliferation Inhibition of Chronic Myeloid Leukemia. Cell Physiol Biochem 47, 981–993. 10.1159/000490142 29843123

[B37] Cheng J. Q. Ruggeri B. Klein W. M. Sonoda G. Altomare D. A. Watson D. K. (1996). Amplification of AKT2 in Human Pancreatic Cells and Inhibition of AKT2 Expression and Tumorigenicity by Antisense RNA. Proc. Natl. Acad. Sci. 93, 3636–3641. 10.1073/pnas.93.8.3636 8622988 PMC39663

[B38] Chini C. C. S. Guerrico A. M. G. Nin V. Camacho-Pereira J. Escande C. Barbosa M. T. (2014). Targeting of NAD Metabolism in Pancreatic Cancer Cells: Potential Novel Therapy for Pancreatic Tumors. Clin. Cancer Res. 20, 120–130. 10.1158/1078-0432.ccr-13-0150 24025713 PMC3947324

[B39] Ciernikova S. Earl J. García Bermejo M. L. Stevurkova V. Carrato A. Smolkova B. (2020). Epigenetic Landscape in Pancreatic Ductal Adenocarcinoma: On the Way to Overcoming Drug Resistance. Ijms 21, 4091. 10.3390/ijms21114091 PMC7311973 32521716

[B40] Clevers H. Loh K. M. Nusse R. (2014). An Integral Program for Tissue Renewal and Regeneration: Wnt Signaling and Stem Cell Control. Science 346, 1248012. 10.1126/science.1248012 25278615

[B41] Collisson E. A. Sadanandam A. Olson P. Gibb W. J. Truitt M. Gu S. (2011). Subtypes of Pancreatic Ductal Adenocarcinoma and Their Differing Responses to Therapy. Nat. Med. 17, 500–503. 10.1038/nm.2344 21460848 PMC3755490

[B42] Collisson E. A. Trejo C. L. Silva J. M. Gu S. Korkola J. E. Heiser L. M. (2012). A Central Role for RAF→MEK→ERK Signaling in the Genesis of Pancreatic Ductal Adenocarcinoma. Cancer Discov. 2, 685–693. 10.1158/2159-8290.cd-11-0347 22628411 PMC3425446

[B43] Commisso C. Davidson S. M. Soydaner-Azeloglu R. G. Parker S. J. Kamphorst J. J. Hackett S. (2013). Macropinocytosis of Protein Is an Amino Acid Supply Route in Ras-Transformed Cells. Nature 497, 633–637. 10.1038/nature12138 23665962 PMC3810415

[B44] Conroy T. Desseigne F. Ychou M. Bouché O. Guimbaud R. Bécouarn Y. (2011). FOLFIRINOX versus Gemcitabine for Metastatic Pancreatic Cancer. N. Engl. J. Med. 364, 1817–1825. 10.1056/nejmoa1011923 21561347

[B45] Conway J. R. Herrmann D. Evans T. J. Morton J. P. Timpson P. (2019). Combating Pancreatic Cancer with PI3K Pathway Inhibitors in the Era of Personalised Medicine. Gut 68, 742–758. 10.1136/gutjnl-2018-316822 30396902 PMC6580874

[B46] Cox A. D. Fesik S. W. Kimmelman A. C. Luo J. Der C. J. (2014). Drugging the Undruggable RAS: Mission Possible. Nat. Rev. Drug Discov. 13, 828–851. 10.1038/nrd4389 25323927 PMC4355017

[B47] Currie R. A. Walker K. S. Gray A. Deak M. Casamayor M. Downes C. P. (1999). Role of Phosphatidylinositol 3,4,5-trisphosphate in Regulating the Activity and Localization of 3-phosphoinositide-dependent Protein Kinase-1. Biochem. J. 337, 575–583. 10.1042/bj3370575 9895304 PMC1220012

[B48] Daemen A. Peterson D. Sahu N. McCord R. Du X. Liu B. (2015). Metabolite Profiling Stratifies Pancreatic Ductal Adenocarcinomas into Subtypes with Distinct Sensitivities to Metabolic Inhibitors. Proc. Natl. Acad. Sci. USA 112, E4410–E4417. 10.1073/pnas.1501605112 26216984 PMC4538616

[B49] Dardare J. Witz A. Merlin J.-L. Gilson P. Harlé A. (2020). SMAD4 and the TGFβ Pathway in Patients with Pancreatic Ductal Adenocarcinoma. Ijms 21, 3534. 10.3390/ijms21103534 PMC7278913 32429474

[B50] Darvin P. Toor S. M. Sasidharan Nair V. Elkord E. (2018). Immune Checkpoint Inhibitors: Recent Progress and Potential Biomarkers. Exp. Mol. Med. 50, 1–11. 10.1038/s12276-018-0191-1 PMC6292890 30546008

[B51] David C. J. Huang Y.-H. Chen M. Su J. Zou Y. Bardeesy N. (2016). TGF-β Tumor Suppression through a Lethal EMT. Cell 164, 1015–1030. 10.1016/j.cell.2016.01.009 26898331 PMC4801341

[B52] Di Nicolantonio F. Martini M. Molinari F. Sartore-Bianchi A. Arena S. Saletti P. (2008). Wild-Type BRAF Is Required for Response to Panitumumab or Cetuximab in Metastatic Colorectal Cancer. Jco 26, 5705–5712. 10.1200/jco.2008.18.0786 19001320

[B53] Diaz L. Marabelle A. Kim T. W. Geva R. Van Cutsem E. André T. (2017). Efficacy of Pembrolizumab in Phase 2 KEYNOTE-164 and KEYNOTE-158 Studies of Microsatellite Instability High Cancers. Ann. Oncol. 28, v128–v129. 10.1093/annonc/mdx367.020

[B54] Driehuis E. van Hoeck A. Moore K. Kolders S. Francies H. E. Gulersonmez M. C. (2019). Pancreatic Cancer Organoids Recapitulate Disease and Allow Personalized Drug Screening. Proc. Natl. Acad. Sci. USA 116, 26580–26590. 10.1073/pnas.1911273116 PMC6936689 31818951

[B55] Drost J. Clevers H. (2018). Organoids in Cancer Research. Nat. Rev. Cancer 18, 407–418. 10.1038/s41568-018-0007-6 29692415

[B56] Duluc C. Moatassim‐Billah S. Chalabi‐Dchar M. Perraud A. Samain R. Breibach F. (2015). Pharmacological Targeting of the Protein Synthesis mTOR/4E‐ BP 1 Pathway in Cancer‐associated Fibroblasts Abrogates Pancreatic Tumour Chemoresistance. Embo Mol. Med. 7, 735–753. 10.15252/emmm.201404346 25834145 PMC4459815

[B57] Dummer R. Ascierto P. A. Gogas H. J. Arance A. Mandala M. Liszkay G. (2018). Overall Survival in Patients with BRAF-Mutant Melanoma Receiving Encorafenib Plus Binimetinib versus Vemurafenib or Encorafenib (COLUMBUS): a Multicentre, Open-Label, Randomised, Phase 3 Trial. Lancet Oncol. 19, 1315–1327. 10.1016/s1470-2045(18)30497-2 30219628

[B58] Eberle J. (2019). Countering TRAIL Resistance in Melanoma. Cancers 11, 656. 10.3390/cancers11050656 PMC6562618 31083589

[B59] Edling C. E. Selvaggi F. Buus R. Maffucci T. Di Sebastiano P. Friess H. (2010). Key Role of Phosphoinositide 3-Kinase Class IB in Pancreatic Cancer. Clin. Cancer Res. 16, 4928–4937. 10.1158/1078-0432.ccr-10-1210 20876794

[B60] Engelman J. A. Chen L. Tan X. Crosby K. Guimaraes A. R. Upadhyay R. (2008). Effective Use of PI3K and MEK Inhibitors to Treat Mutant Kras G12D and PIK3CA H1047R Murine Lung Cancers. Nat. Med. 14, 1351–1356. 10.1038/nm.1890 19029981 PMC2683415

[B61] Eser S. Reiff N. Messer M. Seidler B. Gottschalk K. Dobler M. (2013). Selective Requirement of PI3K/PDK1 Signaling for Kras Oncogene-Driven Pancreatic Cell Plasticity and Cancer. Cancer Cell 23, 406–420. 10.1016/j.ccr.2013.01.023 23453624

[B62] Eser S. Schnieke A. Schneider G. Saur D. (2014). Oncogenic KRAS Signalling in Pancreatic Cancer. Br. J. Cancer 111, 817–822. 10.1038/bjc.2014.215 24755884 PMC4150259

[B63] Eso Y. Shimizu T. Takeda H. Takai A. Marusawa H. (2020). Microsatellite Instability and Immune Checkpoint Inhibitors: toward Precision Medicine against Gastrointestinal and Hepatobiliary Cancers. J. Gastroenterol. 55, 15–26. 10.1007/s00535-019-01620-7 31494725 PMC6942585

[B64] Esteller M. Garcia-Foncillas J. Andion E. Goodman S. N. Hidalgo O. F. Vanaclocha V. (2000). Inactivation of the DNA-Repair GeneMGMTand the Clinical Response of Gliomas to Alkylating Agents. N. Engl. J. Med. 343, 1350–1354. 10.1056/nejm200011093431901 11070098

[B65] Fang Y. Su Z. Xie J. Xue R. Ma Q. Li Y. (2017). Genomic Signatures of Pancreatic Adenosquamous Carcinoma (PASC). J. Pathol. 243, 155–159. 10.1002/path.4943 28722109

[B66] Fischer M. M. Cancilla B. Yeung V. P. Cattaruzza F. Chartier C. Murriel C. L. (2017). WNT Antagonists Exhibit Unique Combinatorial Antitumor Activity with Taxanes by Potentiating Mitotic Cell Death. Sci. Adv. 3, e1700090. 10.1126/sciadv.1700090 28691093 PMC5479655

[B67] Flaherty K. T. Infante J. R. Daud A. Gonzalez R. Kefford R. F. Sosman J. (2012). Combined BRAF and MEK Inhibition in Melanoma with BRAF V600 Mutations. N. Engl. J. Med. 367, 1694–1703. 10.1056/nejmoa1210093 23020132 PMC3549295

[B68] Foggetti G. Li C. Cai H. Hellyer J. A. Lin W.-Y. Ayeni D. (2021). Genetic Determinants of EGFR-Driven Lung Cancer Growth and Therapeutic Response *In Vivo* . Cancer Discov. 11, 1736–1753. 10.1158/2159-8290.cd-20-1385 33707235 PMC8530463

[B69] Foo W. C. Rashid A. Wang H. Katz M. H. Lee J. E. Pisters P. W. (2013). Loss of Phosphatase and Tensin Homolog Expression Is Associated with Recurrence and Poor Prognosis in Patients with Pancreatic Ductal Adenocarcinoma. Hum. Pathol. 44, 1024–1030. 10.1016/j.humpath.2012.09.001 23260327 PMC3832989

[B70] Foster S. A. Whalen D. M. Özen A. Wongchenko M. J. Yin J. Yen I. (2016). Activation Mechanism of Oncogenic Deletion Mutations in BRAF, EGFR, and HER2. Cancer Cell 29, 477–493. 10.1016/j.ccell.2016.02.010 26996308

[B71] Fujii M. Shimokawa M. Date S. Takano A. Matano M. Nanki K. (2016). A Colorectal Tumor Organoid Library Demonstrates Progressive Loss of Niche Factor Requirements during Tumorigenesis. Cell Stem Cell 18, 827–838. 10.1016/j.stem.2016.04.003 27212702

[B72] Fukushima N. Sato N. Ueki T. Rosty C. Walter K. M. Wilentz R. E. (2002). Aberrant Methylation of Preproenkephalin and P16 Genes in Pancreatic Intraepithelial Neoplasia and Pancreatic Ductal Adenocarcinoma. Am. J. Pathol. 160, 1573–1581. 10.1016/s0002-9440(10)61104-2 12000709 PMC1850889

[B73] Gao D. Vela I. Sboner A. Iaquinta P. J. Karthaus W. R. Gopalan A. (2014). Organoid Cultures Derived from Patients with Advanced Prostate Cancer. Cell 159, 176–187. 10.1016/j.cell.2014.08.016 25201530 PMC4237931

[B74] Gazin C. Wajapeyee N. Gobeil S. Virbasius C.-M. Green M. R. (2007). An Elaborate Pathway Required for Ras-Mediated Epigenetic Silencing. Nature 449, 1073–1077. 10.1038/nature06251 17960246 PMC2147719

[B75] Glazer E. S. Welsh E. Pimiento J. M. Teer J. K. Malafa M. P. (2017). TGFβ1 Overexpression Is Associated with Improved Survival and Low Tumor Cell Proliferation in Patients with Early-Stage Pancreatic Ductal Adenocarcinoma. Oncotarget 8, 999–1006. 10.18632/oncotarget.13533 27895310 PMC5352213

[B76] Golan T. Hammel P. Reni M. Van Cutsem E. Macarulla T. Hall M. J. (2019). Maintenance Olaparib for Germline BRCA-Mutated Metastatic Pancreatic Cancer. N. Engl. J. Med. 381, 317–327. 10.1056/nejmoa1903387 31157963 PMC6810605

[B77] Gomez-Puerto M. C. Iyengar P. V. García de Vinuesa A. ten Dijke P. Sanchez-Duffhues G. (2019). Bone Morphogenetic Protein Receptor Signal Transduction in Human Disease. J. Pathol. 247, 9–20. 10.1002/path.5170 30246251 PMC6587955

[B78] Gottesman M. M. Fojo T. Bates S. E. (2002). Multidrug Resistance in Cancer: Role of ATP-dependent Transporters. Nat. Rev. Cancer 2, 48–58. 10.1038/nrc706 11902585

[B79] Graupera M. Guillermet-Guibert J. Foukas L. C. Phng L.-K. Cain R. J. Salpekar A. (2008). Angiogenesis Selectively Requires the P110α Isoform of PI3K to Control Endothelial Cell Migration. Nature 453, 662–666. 10.1038/nature06892 18449193

[B80] Grigson E. R. Ozerova M. Pisklakova A. Liu H. Sullivan D. M. Nefedova Y. (2015). Canonical Wnt Pathway Inhibitor ICG-001 Induces Cytotoxicity of Multiple Myeloma Cells in Wnt-independent Manner. Plos One 10, e0117693. 10.1371/journal.pone.0117693 25635944 PMC4311909

[B81] Guillaumond F. Bidaut G. Ouaissi M. Servais S. Gouirand V. Olivares O. (2015). Cholesterol Uptake Disruption, in Association with Chemotherapy, Is a Promising Combined Metabolic Therapy for Pancreatic Adenocarcinoma. Proc. Natl. Acad. Sci. USA 112, 2473–2478. 10.1073/pnas.1421601112 25675507 PMC4345573

[B82] Guillaumond F. Leca J. Olivares O. Lavaut M.-N. Vidal N. Berthezène P. (2013). Strengthened Glycolysis under Hypoxia Supports Tumor Symbiosis and Hexosamine Biosynthesis in Pancreatic Adenocarcinoma. Proc. Natl. Acad. Sci. 110, 3919–3924. 10.1073/pnas.1219555110 23407165 PMC3593894

[B83] Guo J. Y. Chen H.-Y. Mathew R. Fan J. Strohecker A. M. Karsli-Uzunbas G. (2011). Activated Ras Requires Autophagy to Maintain Oxidative Metabolism and Tumorigenesis. Genes Develop. 25, 460–470. 10.1101/gad.2016311 21317241 PMC3049287

[B84] Gurney A. Axelrod F. Bond C. J. Cain J. Chartier C. Donigan L. (2012). Wnt Pathway Inhibition via the Targeting of Frizzled Receptors Results in Decreased Growth and Tumorigenicity of Human Tumors. Proc. Natl. Acad. Sci. 109, 11717–11722. 10.1073/pnas.1120068109 22753465 PMC3406803

[B85] Hahn S. A. Schutte M. Hoque A. T. M. S. Moskaluk C. A. da Costa L. T. Rozenblum E. (1996). DPC4, A Candidate Tumor Suppressor Gene at Human Chromosome 18q21.1. Science 271, 350–353. 10.1126/science.271.5247.350 8553070

[B86] Hamdan F. H. Johnsen S. A. (2018). DeltaNp63-dependent Super Enhancers Define Molecular Identity in Pancreatic Cancer by an Interconnected Transcription Factor Network. Proc. Natl. Acad. Sci. USA 115, E12343–E12352. 10.1073/pnas.1812915116 30541891 PMC6310858

[B87] Haque S. Morris J. C. (2017). Transforming Growth Factor-β: A Therapeutic Target for Cancer. Hum. Vaccin. Immunother. 13, 1741–1750. 10.1080/21645515.2017.1327107 28575585 PMC5557219

[B88] Hatzivassiliou G. Song K. Yen I. Brandhuber B. J. Anderson D. J. Alvarado R. (2010). RAF Inhibitors Prime Wild-type RAF to Activate the MAPK Pathway and Enhance Growth. Nature 464, 431–435. 10.1038/nature08833 20130576

[B89] Hawkins P. T. Anderson K. E. Davidson K. Stephens L. R. (2006). Signalling through Class I PI3Ks in Mammalian Cells. Biochem. Soc. T 34, 647–662. 10.1042/bst0340647 17052169

[B90] Hayashi A. Fan J. Chen R. Ho Y.-j. Makohon-Moore A. P. Lecomte N. (2020). A Unifying Paradigm for Transcriptional Heterogeneity and Squamous Features in Pancreatic Ductal Adenocarcinoma. Nat. Cancer 1, 59–74. 10.1038/s43018-019-0010-1 PMC8809486 35118421

[B91] Hayes T. K. Neel N. F. Hu C. Gautam P. Chenard M. Long B. (2016). Long-Term ERK Inhibition in KRAS-Mutant Pancreatic Cancer Is Associated with MYC Degradation and Senescence-like Growth Suppression. Cancer Cell 29, 75–89. 10.1016/j.ccell.2015.11.011 26725216 PMC4816652

[B92] Hegi M. E. Diserens A.-C. Gorlia T. Hamou M.-F. de Tribolet N. Weller M. (2005). MGMTGene Silencing and Benefit from Temozolomide in Glioblastoma. N. Engl. J. Med. 352, 997–1003. 10.1056/nejmoa043331 15758010

[B93] Heidenblad M. Jonson T. Mahlamäki E. H. Gorunova L. Karhu R. Johansson B. (2002). Detailed Genomic Mapping and Expression Analyses of 12p Amplifications in Pancreatic Carcinomas Reveal a 3.5-Mb Target Region for Amplification. Genes Chromosom. Cancer 34, 211–223. 10.1002/gcc.10063 11979555

[B94] Henriksen A. Dyhl-Polk A. Chen I. Nielsen D. (2019). Checkpoint Inhibitors in Pancreatic Cancer. Cancer Treat. Rev. 78, 17–30. 10.1016/j.ctrv.2019.06.005 31325788

[B95] Hessmann E. Johnsen S. A. Siveke J. T. Ellenrieder V. (2017). Epigenetic Treatment of Pancreatic Cancer: Is There a Therapeutic Perspective on the Horizon. Gut 66, 168–179. 10.1136/gutjnl-2016-312539 27811314 PMC5256386

[B96] Hilmi M. Bartholin L. Neuzillet C. (2018). Immune Therapies in Pancreatic Ductal Adenocarcinoma: Where Are We Now. Wjg 24, 2137–2151. 10.3748/wjg.v24.i20.2137 29853732 PMC5974576

[B97] Hingorani S. R. Wang L. Multani A. S. Combs C. Deramaudt T. B. Hruban R. H. (2005). Trp53R172H and KrasG12D Cooperate to Promote Chromosomal Instability and Widely Metastatic Pancreatic Ductal Adenocarcinoma in Mice. Cancer Cell 7, 469–483. 10.1016/j.ccr.2005.04.023 15894267

[B98] Hobbs G. A. Baker N. M. Miermont A. M. Thurman R. D. Pierobon M. Tran T. H. (2020). Atypical KRASG12R Mutant Is Impaired in PI3K Signaling and Macropinocytosis in Pancreatic Cancer. Cancer Discov. 10, 104–123. 10.1158/2159-8290.cd-19-1006 31649109 PMC6954322

[B99] Hosein A. N. Brekken R. A. Maitra A. (2020). Pancreatic Cancer Stroma: an Update on Therapeutic Targeting Strategies. Nat. Rev. Gastroenterol. Hepatol. 17, 487–505. 10.1038/s41575-020-0300-1 32393771 PMC8284850

[B100] Hu Y. Guo R. Wei J. Zhou Y. Ji W. Liu J. (2015). Effects of PI3K Inhibitor NVP-Bkm120 on Overcoming Drug Resistance and Eliminating Cancer Stem Cells in Human Breast Cancer Cells. Cell Death Dis 6, e2020. 10.1038/cddis.2015.363 26673665 PMC4720896

[B101] Hu Z. I. Shia J. Stadler Z. K. Varghese A. M. Capanu M. Salo-Mullen E. (2018). Evaluating Mismatch Repair Deficiency in Pancreatic Adenocarcinoma: Challenges and Recommendations. Clin. Cancer Res. 24, 1326–1336. 10.1158/1078-0432.ccr-17-3099 29367431 PMC5856632

[B102] Huang B. Z. Chang J. I. Li E. Xiang A. H. Wu B. U. (2016). Influence of Statins and Cholesterol on Mortality Among Patients with Pancreatic Cancer. JNCI J. Natl. Cancer Inst. 109, djw275. 10.1093/jnci/djw275 28040693

[B103] Huang L. Holtzinger A. Jagan I. BeGora M. Lohse I. Ngai N. (2015). Ductal Pancreatic Cancer Modeling and Drug Screening Using Human Pluripotent Stem Cell- and Patient-Derived Tumor Organoids. Nat. Med. 21, 1364–1371. 10.1038/nm.3973 26501191 PMC4753163

[B104] Hussain S. M. Reed L. F. Krasnick B. A. Miranda-Carboni G. Fields R. C. Bi Y. (2018). IL23 and TGF-SS Diminish Macrophage Associated Metastasis in Pancreatic Carcinoma. Sci. Rep. 8, 5808. 10.1038/s41598-018-24194-5 29643359 PMC5895618

[B105] Iacobuzio-Donahue C. A. Velculescu V. E. Wolfgang C. L. Hruban R. H. (2012). Genetic Basis of Pancreas Cancer Development and Progression: Insights from Whole-Exome and Whole-Genome Sequencing. Clin. Cancer Res. 18, 4257–4265. 10.1158/1078-0432.ccr-12-0315 22896692 PMC3422771

[B106] Janes M. R. Zhang J. Li L.-S. Hansen R. Peters U. Guo X. (2018). Targeting KRAS Mutant Cancers with a Covalent G12C-specific Inhibitor. Cell 172, 578–589. e17. 10.1016/j.cell.2018.01.006 29373830

[B107] Janku F. (2017). Phosphoinositide 3-kinase (PI3K) Pathway Inhibitors in Solid Tumors: From Laboratory to Patients. Cancer Treat. Rev. 59, 93–101. 10.1016/j.ctrv.2017.07.005 28779636

[B108] Javle M. M. Shroff R. T. Xiong H. Varadhachary G. A. Fogelman D. Reddy S. A. (2010). Inhibition of the Mammalian Target of Rapamycin (mTOR) in Advanced Pancreatic Cancer: Results of Two Phase II Studies. Bmc Cancer 10, 368. 10.1186/1471-2407-10-368 20630061 PMC2910694

[B109] Jia Y. Xie J. (2015). Promising Molecular Mechanisms Responsible for Gemcitabine Resistance in Cancer. Genes Dis. 2, 299–306. 10.1016/j.gendis.2015.07.003 30258872 PMC6150077

[B110] Jiang H. Li Q. He C. Li F. Sheng H. Shen X. (2014). Activation of the Wnt Pathway through Wnt2 Promotes Metastasis in Pancreatic Cancer. Am. J. Cancer Res. 4, 537–544. 25232495 PMC4163618

[B111] Jiang X. Hao H.-X. Growney J. D. Woolfenden S. Bottiglio C. Ng N. (2013). Inactivating Mutations of RNF43 Confer Wnt Dependency in Pancreatic Ductal Adenocarcinoma. Proc. Natl. Acad. Sci. 110, 12649–12654. 10.1073/pnas.1307218110 23847203 PMC3732970

[B112] Jimeno A. Tan A. C. Coffa J. Rajeshkumar N. V. Kulesza P. Rubio-Viqueira B. (2008). Coordinated Epidermal Growth Factor Receptor Pathway Gene Overexpression Predicts Epidermal Growth Factor Receptor Inhibitor Sensitivity in Pancreatic Cancer. Cancer Res. 68, 2841–2849. 10.1158/0008-5472.can-07-5200 18413752

[B113] Jones P. A. Baylin S. B. (2007). The Epigenomics of Cancer. Cell 128, 683–692. 10.1016/j.cell.2007.01.029 17320506 PMC3894624

[B114] Jones P. A. Baylin S. B. (2002). The Fundamental Role of Epigenetic Events in Cancer. Nat. Rev. Genet. 3, 415–428. 10.1038/nrg816 12042769

[B115] Juiz N. A. Iovanna J. Dusetti N. (2019). Pancreatic Cancer Heterogeneity Can Be Explained beyond the Genome. Front. Oncol. 9, 246. 10.3389/fonc.2019.00246 31024848 PMC6460948

[B116] Juiz N. Elkaoutari A. Bigonnet M. Gayet O. Roques J. Nicolle R. (2020). Basal-like and Classical Cells Coexistence in Pancreatic Cancer Revealed by Single Cell Analysis. FASEB J., 34, 12214–12228. 10.1101/2020.01.07.897454 32686876

[B117] Junttila M. R. Devasthali V. Cheng J. H. Castillo J. Metcalfe C. Clermont A. C. (2015). Modeling Targeted Inhibition of MEK and PI3 Kinase in Human Pancreatic Cancer. Mol. Cancer Ther. 14, 40–47. 10.1158/1535-7163.mct-14-0030 25376606

[B118] Karapetis C. S. Khambata-Ford S. Jonker D. J. O'Callaghan C. J. Tu D. Tebbutt N. C. (2008). K-rasMutations and Benefit from Cetuximab in Advanced Colorectal Cancer. N. Engl. J. Med. 359, 1757–1765. 10.1056/nejmoa0804385 18946061

[B119] Karnoub A. E. Weinberg R. A. (2008). Ras Oncogenes: Split Personalities. Nat. Rev. Mol. Cel Biol 9, 517–531. 10.1038/nrm2438 PMC3915522 18568040

[B120] Karreth F. A. Frese K. K. DeNicola G. M. Baccarini M. Tuveson D. A. (2011). C-raf Is Required for the Initiation of Lung Cancer by K-RasG12D. Cancer Discov. 1, 128–136. 10.1158/2159-8290.cd-10-0044 22043453 PMC3203527

[B121] Kashatus J. A. Nascimento A. Myers L. J. Sher A. Byrne F. L. Hoehn K. L. (2015). Erk2 Phosphorylation of Drp1 Promotes Mitochondrial Fission and MAPK-Driven Tumor Growth. Mol. Cel 57, 537–551. 10.1016/j.molcel.2015.01.002 PMC4393013 25658205

[B122] Kennedy A. L. Morton J. P. Manoharan I. Nelson D. M. Jamieson N. B. Pawlikowski J. S. (2011). Activation of the PIK3CA/AKT Pathway Suppresses Senescence Induced by an Activated RAS Oncogene to Promote Tumorigenesis. Mol. Cel 42, 36–49. 10.1016/j.molcel.2011.02.020 PMC3145340 21474066

[B123] Kerr E. M. Gaude E. Turrell F. K. Frezza C. Martins C. P. (2016). Mutant Kras Copy Number Defines Metabolic Reprogramming and Therapeutic Susceptibilities. Nature 531, 110–113. 10.1038/nature16967 26909577 PMC4780242

[B124] Kinsey C. G. Camolotto S. A. Boespflug A. M. Guillen K. P. Foth M. Truong A. (2019). Protective Autophagy Elicited by RAF→MEK→ERK Inhibition Suggests a Treatment Strategy for RAS-Driven Cancers. Nat. Med. 25, 620–627. 10.1038/s41591-019-0367-9 30833748 PMC6452642

[B125] Kisiel J. B. Raimondo M. Taylor W. R. Yab T. C. Mahoney D. W. Sun Z. (2015). New DNA Methylation Markers for Pancreatic Cancer: Discovery, Tissue Validation, and Pilot Testing in Pancreatic Juice. Clin. Cancer Res. 21, 4473–4481. 10.1158/1078-0432.ccr-14-2469 26023084 PMC4592385

[B126] Klomp J. E. Klomp J. A. Der C. J. (2021). The ERK Mitogen-Activated Protein Kinase Signaling Network: the Final Frontier in RAS Signal Transduction. Biochem. Soc. T 49, 253–267. 10.1042/bst20200507 PMC12691160 33544118

[B127] Knudsen E. S. Witkiewicz A. K. (2017). The Strange Case of CDK4/6 Inhibitors: Mechanisms, Resistance, and Combination Strategies. Trends Cancer 3, 39–55. 10.1016/j.trecan.2016.11.006 28303264 PMC5347397

[B128] Ko A. H. Chiorean E. G. Kwak E. L. Lenz H.-J. Nadler P. I. Wood D. L. (2016). Final Results of a Phase Ib Dose-Escalation Study of PRI-724, a CBP/beta-catenin Modulator, Plus Gemcitabine (GEM) in Patients with Advanced Pancreatic Adenocarcinoma (APC) as Second-Line Therapy after FOLFIRINOX or FOLFOX. Jco 34, e15721. 10.1200/jco.2016.34.15_suppl.e15721

[B129] Kondo J. Endo H. Okuyama H. Ishikawa O. Iishi H. Tsujii M. (2011). Retaining Cell-Cell Contact Enables Preparation and Culture of Spheroids Composed of Pure Primary Cancer Cells from Colorectal Cancer. Proc. Natl. Acad. Sci. 108, 6235–6240. 10.1073/pnas.1015938108 21444794 PMC3076886

[B130] Krishnamurthy N. Kurzrock R. (2018). Targeting the Wnt/beta-Catenin Pathway in Cancer: Update on Effectors and Inhibitors. Cancer Treat. Rev. 62, 50–60. 10.1016/j.ctrv.2017.11.002 29169144 PMC5745276

[B131] Kumar R. Chaudhary K. Gupta S. Singh H. Kumar S. Gautam A. (2013). CancerDR: Cancer Drug Resistance Database. Sci. Rep. 3, 1445. 10.1038/srep01445 23486013 PMC3595698

[B132] Lamba S. Russo M. Sun C. Lazzari L. Cancelliere C. Grernrum W. (2014). RAF Suppression Synergizes with MEK Inhibition in KRAS Mutant Cancer Cells. Cel Rep. 8, 1475–1483. 10.1016/j.celrep.2014.07.033 25199829

[B133] Larkin J. Ascierto P. A. Dréno B. Atkinson V. Liszkay G. Maio M. (2014). Combined Vemurafenib and Cobimetinib in BRAF-Mutated Melanoma. N. Engl. J. Med. 371, 1867–1876. 10.1056/nejmoa1408868 25265494

[B134] Lemmon M. A. Schlessinger J. (2010). Cell Signaling by Receptor Tyrosine Kinases. Cell 141, 1117–1134. 10.1016/j.cell.2010.06.011 20602996 PMC2914105

[B135] Lenz H. J. Kahn M. (2014). Safely Targeting Cancer Stem Cells via Selective Catenin Coactivator Antagonism. Cancer Sci. 105, 1087–1092. 10.1111/cas.12471 24975284 PMC4175086

[B136] Li D. Xie K. Wolff R. Abbruzzese J. L. (2004). Pancreatic Cancer. The Lancet 363, 1049–1057. 10.1016/s0140-6736(04)15841-8 15051286

[B137] Liang C. Wang Z. Li Y.-Y. Yu B.-H. Zhang F. Li H.-Y. (2015). miR-33a Suppresses the Nuclear Translocation of β-catenin to Enhance Gemcitabine Sensitivity in Human Pancreatic Cancer Cells. Tumor Biol. 36, 9395–9403. 10.1007/s13277-015-3679-5 26113407

[B138] Lito P. Solomon M. Li L.-S. Hansen R. Rosen N. (2016). Allele-specific Inhibitors Inactivate Mutant KRAS G12C by a Trapping Mechanism. Science 351, 604–608. 10.1126/science.aad6204 26841430 PMC4955282

[B139] Liu M.-L. Lintig F. C. V. Liyanage M. Shibata M.-A. Jorcyk C. L. Ried T. (1998). Amplification of Ki-Ras and Elevation of MAP Kinase Activity during Mammary Tumor Progression in C3(1)/SV40 Tag Transgenic Mice. Oncogene 17, 2403–2411. 10.1038/sj.onc.1202456 9811472

[B140] Liu R. Chen Y. Liu G. Li C. Song Y. Cao Z. (2020). PI3K/AKT Pathway as a Key Link Modulates the Multidrug Resistance of Cancers. Cel Death Dis 11, 797. 10.1038/s41419-020-02998-6 PMC7515865 32973135

[B141] Lomberk G. Blum Y. Nicolle R. Nair A. Gaonkar K. S. Marisa L. (2018). Distinct Epigenetic Landscapes Underlie the Pathobiology of Pancreatic Cancer Subtypes. Nat. Commun. 9, 1978. 10.1038/s41467-018-04383-6 29773832 PMC5958058

[B142] Long G. V. Stroyakovskiy D. Gogas H. Levchenko E. de Braud F. Larkin J. (2014). Combined BRAF and MEK Inhibition versus BRAF Inhibition Alone in Melanoma. N. Engl. J. Med. 371, 1877–1888. 10.1056/nejmoa1406037 25265492

[B143] LoPiccolo J. Blumenthal G. Bernstein W. Dennis P. (2008). Targeting the PI3K/Akt/mTOR Pathway: Effective Combinations and Clinical Considerations. Drug Resist. Updates 11, 32–50. 10.1016/j.drup.2007.11.003 PMC2442829 18166498

[B144] Lu W. Hu Y. Chen G. Chen Z. Zhang H. Wang F. (2012). Novel Role of NOX in Supporting Aerobic Glycolysis in Cancer Cells with Mitochondrial Dysfunction and as a Potential Target for Cancer Therapy. Plos Biol. 10, e1001326. 10.1371/journal.pbio.1001326 22589701 PMC3348157

[B145] Luchini C. Paolino G. Mattiolo P. Piredda M. L. Cavaliere A. Gaule M. (2020). KRAS Wild-type Pancreatic Ductal Adenocarcinoma: Molecular Pathology and Therapeutic Opportunities. J. Exp. Clin. Cancer Res. 39, 227. 10.1186/s13046-020-01732-6 33115526 PMC7594413

[B146] Maehama T. Dixon J. E. (1998). The Tumor Suppressor, PTEN/MMAC1, Dephosphorylates the Lipid Second Messenger, Phosphatidylinositol 3,4,5-Trisphosphate. J. Biol. Chem. 273, 13375–13378. 10.1074/jbc.273.22.13375 9593664

[B147] Manegold P. Lai K. Wu Y. Teo J.-L. Lenz H.-J. Genyk Y. (2018). Differentiation Therapy Targeting the β-Catenin/CBP Interaction in Pancreatic Cancer. Cancers 10, 95. 10.3390/cancers10040095 PMC5923350 29596326

[B148] Massagué J. (2008). TGFβ in Cancer. Cell 134, 215–230. 10.1016/j.cell.2008.07.001 18662538 PMC3512574

[B149] Matsubayashi H. Canto M. Sato N. Klein A. Abe T. Yamashita K. (2006). DNA Methylation Alterations in the Pancreatic Juice of Patients with Suspected Pancreatic Disease. Cancer Res. 66, 1208–1217. 10.1158/0008-5472.can-05-2664 16424060

[B150] Melzer C. Hass R. von der Ohe J. Lehnert H. Ungefroren H. (2017). The Role of TGF-β and its Crosstalk with RAC1/RAC1b Signaling in Breast and Pancreas Carcinoma. Cell Commun Signal 15, 19. 10.1186/s12964-017-0175-0 28499439 PMC5429551

[B151] Miao C.-g. Yang Y.-y. He X. Huang C. Huang Y. Zhang L. (2013). Wnt Signaling in Liver Fibrosis: Progress, Challenges and Potential Directions. Biochimie 95, 2326–2335. 10.1016/j.biochi.2013.09.003 24036368

[B152] Milroy L.-G. Ottmann C. (2014). The Renaissance of Ras. ACS Chem. Biol. 9, 2447–2458. 10.1021/cb500555h 25148694

[B153] Miyabayashi K. Baker L. A. Deschênes A. Traub B. Caligiuri G. Plenker D. (2020). Intraductal Transplantation Models of Human Pancreatic Ductal Adenocarcinoma Reveal Progressive Transition of Molecular Subtypes. Cancer Discov. 10, 1566–1589. 10.1158/2159-8290.cd-20-0133 32703770 PMC7664990

[B154] Modi S. Kir D. Banerjee S. Saluja A. (2016). Control of Apoptosis in Treatment and Biology of Pancreatic Cancer. J. Cel. Biochem. 117, 279–288. 10.1002/jcb.25284 PMC5724757 26206252

[B155] Moffat J. G. Vincent F. Lee J. A. Eder J. Prunotto M. (2017). Opportunities and Challenges in Phenotypic Drug Discovery: an Industry Perspective. Nat. Rev. Drug Discov. 16, 531–543. 10.1038/nrd.2017.111 28685762

[B156] Moffitt R. A. Marayati R. Flate E. L. Volmar K. E. Loeza S. G. H. Hoadley K. A. (2015). Virtual Microdissection Identifies Distinct Tumor- and Stroma-specific Subtypes of Pancreatic Ductal Adenocarcinoma. Nat. Genet. 47, 1168–1178. 10.1038/ng.3398 26343385 PMC4912058

[B157] Moore M. J. Goldstein D. Hamm J. Figer A. Hecht J. R. Gallinger S. (2007). Erlotinib Plus Gemcitabine Compared with Gemcitabine Alone in Patients with Advanced Pancreatic Cancer: A Phase III Trial of the National Cancer Institute of Canada Clinical Trials Group. Jco 25, 1960–1966. 10.1200/jco.2006.07.9525 17452677

[B158] Morel D. Jeffery D. Aspeslagh S. Almouzni G. Postel-Vinay S. (2020). Combining Epigenetic Drugs with Other Therapies for Solid Tumours - Past Lessons and Future Promise. Nat. Rev. Clin. Oncol. 17, 91–107. 10.1038/s41571-019-0267-4 31570827

[B159] Murtaugh L. C. Kopinke D. (2008). Pancreatic Stem Cells. Cambridge, Massachusetts: Stembook. 10.3824/stembook.1.3.1 20614618

[B160] Murtaugh L. C. (2008). The what, where, when and How of Wnt/β-Catenin Signaling in Pancreas Development. Organogenesis 4, 81–86. 10.4161/org.4.2.5853 18953422 PMC2572215

[B161] Muzumdar M. D. Chen P.-Y. Dorans K. J. Chung K. M. Bhutkar A. Hong E. (2017). Survival of Pancreatic Cancer Cells Lacking KRAS Function. Nat. Commun. 8, 1090. 10.1038/s41467-017-00942-5 29061961 PMC5653666

[B162] Nagano H. Tomimaru Y. Eguchi H. Hama N. Wada H. Kawamoto K. (2013). MicroRNA-29a Induces Resistance to Gemcitabine through the Wnt/β-Catenin Signaling Pathway in Pancreatic Cancer Cells. Int. J. Oncol. 43, 1066–1072. 10.3892/ijo.2013.2037 23900458

[B163] Nagpal G. Sharma M. Kumar S. Chaudhary K. Gupta S. Gautam A. (2014). PCMdb: Pancreatic Cancer Methylation Database. Sci. Rep. 4, 4197. 10.1038/srep04197 24569397 PMC3935225

[B164] Nakamura T. Sano M. Songyang Z. Schneider M. D. (2003). A Wnt- and -catenin-dependent Pathway for Mammalian Cardiac Myogenesis. Proc. Natl. Acad. Sci. 100, 5834–5839. 10.1073/pnas.0935626100 12719544 PMC156287

[B165] Nemunaitis J. Jahan T. Ross H. Sterman D. Richards D. Fox B. (2006). Phase 1/2 Trial of Autologous Tumor Mixed with an Allogeneic GVAX Vaccine in Advanced-Stage Non-small-cell Lung Cancer. Cancer Gene Ther. 13, 555–562. 10.1038/sj.cgt.7700922 16410826

[B166] Nicolle R. Blum Y. Marisa L. Loncle C. Gayet O. Moutardier V. (2017). Pancreatic Adenocarcinoma Therapeutic Targets Revealed by Tumor-Stroma Cross-Talk Analyses in Patient-Derived Xenografts. Cel Rep. 21, 2458–2470. 10.1016/j.celrep.2017.11.003 PMC6082139 29186684

[B167] O'Hagan R. C. Chang S. Maser R. S. Mohan R. Artandi S. E. Chin L. (2002). Telomere Dysfunction Provokes Regional Amplification and Deletion in Cancer Genomes. Cancer Cell 2, 149–155. 10.1016/s1535-6108(02)00094-6 12204535

[B168] Oettle H. Hilbig A. Seufferlein T. Tsianakas A. Luger T. Schmid R. M. (2011). Phase I/II Study with Trabedersen (AP 12009) Monotherapy for the Treatment of Patients with Advanced Pancreatic Cancer, Malignant Melanoma, and Colorectal Carcinoma. Jco 29, 2513. 10.1200/jco.2011.29.15_suppl.2513

[B169] Öhlund D. Handly-Santana A. Biffi G. Elyada E. Almeida A. S. Ponz-Sarvise M. (2017). Distinct Populations of Inflammatory Fibroblasts and Myofibroblasts in Pancreatic Cancer. J. Exp. Med. 214, 579–596. 10.1084/jem.20162024 28232471 PMC5339682

[B170] Omura N. Li C.-P. Li A. Hong S.-M. Walter K. Jimeno A. (2008). Genome-wide Profiling at Methylated Promoters in Pancreatic Adenocarcinoma. Cancer Biol. Ther. 7, 1146–1156. 10.4161/cbt.7.7.6208 18535405 PMC2763640

[B171] Orth M. Metzger P. Gerum S. Mayerle J. Schneider G. Belka C. (2019). Pancreatic Ductal Adenocarcinoma: Biological Hallmarks, Current Status, and Future Perspectives of Combined Modality Treatment Approaches. Radiat. Oncol. 14, 141. 10.1186/s13014-019-1345-6 31395068 PMC6688256

[B172] Ostrem J. M. Peters U. Sos M. L. Wells J. A. Shokat K. M. (2013). K-Ras(G12C) Inhibitors Allosterically Control GTP Affinity and Effector Interactions. Nature 503, 548–551. 10.1038/nature12796 24256730 PMC4274051

[B173] Ozkan-Dagliyan I. Diehl J. N. George S. D. Schaefer A. Papke B. Klotz-Noack K. (2020). Low-Dose Vertical Inhibition of the RAF-MEK-ERK Cascade Causes Apoptotic Death of KRAS Mutant Cancers. Cel Rep. 31, 107764. 10.1016/j.celrep.2020.107764 PMC7393480 32553168

[B174] Pasca di Magliano M. Biankin A. V. Heiser P. W. Cano D. A. Gutierrez P. J. A. Deramaudt T. (2007). Common Activation of Canonical Wnt Signaling in Pancreatic Adenocarcinoma. Plos One 2, e1155. 10.1371/journal.pone.0001155 17982507 PMC2048934

[B175] Payne S. N. Maher M. E. Tran N. H. Van De Hey D. R. Foley T. M. Yueh A. E. (2015). PIK3CA Mutations Can Initiate Pancreatic Tumorigenesis and Are Targetable with PI3K Inhibitors. Oncogenesis 4, e169. 10.1038/oncsis.2015.28 26436951 PMC4632089

[B176] Pfohl U. Pflaume A. Regenbrecht M. Finkler S. Graf Adelmann Q. Reinhard C. (2021). Precision Oncology beyond Genomics: The Future Is Here-It Is Just Not Evenly Distributed. Cells 10, 928. 10.3390/cells10040928 33920536 PMC8072767

[B177] Phan N. Hong J. J. Tofig B. Mapua M. Elashoff D. Moatamed N. A. (2019). A Simple High-Throughput Approach Identifies Actionable Drug Sensitivities in Patient-Derived Tumor Organoids. Commun. Biol. 2, 78. 10.1038/s42003-019-0305-x 30820473 PMC6389967

[B178] Pickup M. Novitskiy S. Moses H. L. (2013). The Roles of TGFβ in the Tumour Microenvironment. Nat. Rev. Cancer 13, 788–799. 10.1038/nrc3603 24132110 PMC4025940

[B179] Poulikakos P. I. Zhang C. Bollag G. Shokat K. M. Rosen N. (2010). RAF Inhibitors Transactivate RAF Dimers and ERK Signalling in Cells with Wild-type BRAF. Nature 464, 427–430. 10.1038/nature08902 20179705 PMC3178447

[B180] Principe D. R. Diaz A. M. Torres C. Mangan R. J. DeCant B. McKinney R. (2017). TGFβ Engages MEK/ERK to Differentially Regulate Benign and Malignant Pancreas Cell Function. Oncogene 36, 4336–4348. 10.1038/onc.2016.500 28368414 PMC5537609

[B181] Principe D. R. Doll J. A. Bauer J. Jung B. Munshi H. G. Bartholin L. (2014). TGF- : Duality of Function between Tumor Prevention and Carcinogenesis. JNCI J. Natl. Cancer Inst. 106, djt369. 10.1093/jnci/djt369 24511106 PMC3952197

[B182] Proffitt K. D. Virshup D. M. (2012). Precise Regulation of Porcupine Activity Is Required for Physiological Wnt Signaling. J. Biol. Chem. 287, 34167–34178. 10.1074/jbc.m112.381970 22888000 PMC3464525

[B183] Puca L. Bareja R. Prandi D. Shaw R. Benelli M. Karthaus W. R. (2018). Patient Derived Organoids to Model Rare Prostate Cancer Phenotypes. Nat. Commun. 9, 2404. 10.1038/s41467-018-04495-z 29921838 PMC6008438

[B184] Puleo F. Nicolle R. Blum Y. Cros J. Marisa L. Demetter P. (2018). Stratification of Pancreatic Ductal Adenocarcinomas Based on Tumor and Microenvironment Features. Gastroenterology 155, 1999–2013. e3. 10.1053/j.gastro.2018.08.033 30165049

[B185] Putiri E. L. Robertson K. D. (2011). Epigenetic Mechanisms and Genome Stability. Clin. Epigenet 2, 299–314. 10.1007/s13148-010-0017-z PMC3172155 21927626

[B186] Rahib L. Smith B. D. Aizenberg R. Rosenzweig A. B. Fleshman J. M. Matrisian L. M. (2014). Projecting Cancer Incidence and Deaths to 2030: The Unexpected Burden of Thyroid, Liver, and Pancreas Cancers in the United States. Cancer Res. 74, 2913–2921. 10.1158/0008-5472.can-14-0155 24840647

[B187] Ram Makena M. Gatla H. Verlekar D. Sukhavasi S. K. Pandey M. C. Pramanik K. (2019). Wnt/β-Catenin Signaling: The Culprit in Pancreatic Carcinogenesis and Therapeutic Resistance. Ijms 20, 4242. 10.3390/ijms20174242 PMC6747343 31480221

[B188] Raphael B. J. Hruban R. H. Aguirre A. J. Moffitt R. A. Yeh J. J. Stewart C. (2017). Integrated Genomic Characterization of Pancreatic Ductal Adenocarcinoma. Cancer Cell 32, 185–e13. 10.1016/j.ccell.2017.07.007 28810144 PMC5964983

[B189] Riquelme E. Behrens C. Lin H. Y. Simon G. Papadimitrakopoulou V. Izzo J. (2016). Modulation of EZH2 Expression by MEK-ERK or PI3K-AKT Signaling in Lung Cancer Is Dictated by Different KRAS Oncogene Mutations. Cancer Res. 76, 675–685. 10.1158/0008-5472.can-15-1141 26676756 PMC4738155

[B190] Ruggeri B. A. Huang L. Wood M. Cheng J. Q. Testa J. R. (1998). Amplification and Overexpression of theAKT2 Oncogene in a Subset of Human Pancreatic Ductal Adenocarcinomas. Mol. Carcinog. 21, 81–86. 10.1002/(sici)1098-2744(199802)21:2<81:aid-mc1>3.0.co;2-r 9496907

[B191] Ryan D. P. Hong T. S. Bardeesy N. (2014). Pancreatic Adenocarcinoma. N. Engl. J. Med. 371, 1039–1049. 10.1056/nejmra1404198 25207767

[B192] Sachs N. de Ligt J. Kopper O. Gogola E. Bounova G. Weeber F. (2018). A Living Biobank of Breast Cancer Organoids Captures Disease Heterogeneity. Cell 172, 373–386. e10. 10.1016/j.cell.2017.11.010 29224780

[B193] Saito T. Ishido K. Kudo D. Kimura N. Wakiya T. Nakayama Y. (2017). Combination Therapy with Gemcitabine and Nab-Paclitaxel for Locally Advanced Unresectable Pancreatic Cancer. Mol. Clin. Oncol. 6, 963–967. 10.3892/mco.2017.1251 28588798 PMC5451866

[B194] Saka D. Gökalp M. Piyade B. Cevik N. C. Arik Sever E. Unutmaz D. (2020). Mechanisms of T-Cell Exhaustion in Pancreatic Cancer. Cancers 12, 2274. 10.3390/cancers12082274 PMC7464444 32823814

[B195] Samatar A. A. Poulikakos P. I. (2014). Targeting RAS-ERK Signalling in Cancer: Promises and Challenges. Nat. Rev. Drug Discov. 13, 928–942. 10.1038/nrd4281 25435214

[B196] Sano M. Driscoll D. R. DeJesus-Monge W. E. Quattrochi B. Appleman V. A. Ou J. (2016). Activation of WNT/β-Catenin Signaling Enhances Pancreatic Cancer Development and the Malignant Potential via Up-Regulation of Cyr61. Neoplasia 18, 785–794. 10.1016/j.neo.2016.11.004 27889647 PMC5126137

[B197] Sato N. Fukushima N. Maitra A. Matsubayashi H. Yeo C. J. Cameron J. L. (2003b). Discovery of Novel Targets for Aberrant Methylation in Pancreatic Carcinoma Using High-Throughput Microarrays. Cancer Res. 63, 3735–3742. 12839967

[B198] Sato N. Fukushima N. Maehara N. Matsubayashi H. Koopmann J. Su G. H. (2003a). SPARC/osteonectin Is a Frequent Target for Aberrant Methylation in Pancreatic Adenocarcinoma and a Mediator of Tumor-Stromal Interactions. Oncogene 22, 5021–5030. 10.1038/sj.onc.1206807 12902985

[B199] Sato N. Parker A. R. Fukushima N. Miyagi Y. Iacobuzio-Donahue C. A. Eshleman J. R. (2005). Epigenetic Inactivation of TFPI-2 as a Common Mechanism Associated with Growth and Invasion of Pancreatic Ductal Adenocarcinoma. Oncogene 24, 850–858. 10.1038/sj.onc.1208050 15592528

[B200] Scheffzek K. Ahmadian M. R. Kabsch W. Wiesmüller L. Lautwein A. Schmitz F. (1997). The Ras-RasGAP Complex: Structural Basis for GTPase Activation and its Loss in Oncogenic Ras Mutants. Science 277, 333–339. 10.1126/science.277.5324.333 9219684

[B201] Schlieman M. G. Fahy B. N. Ramsamooj R. Beckett L. Bold R. J. (2003). Incidence, Mechanism and Prognostic Value of Activated AKT in Pancreas Cancer. Br. J. Cancer 89, 2110–2115. 10.1038/sj.bjc.6601396 14647146 PMC2376856

[B202] Schumacher D. Andrieux G. Boehnke K. Keil M. Silvestri A. Silvestrov M. (2019). Heterogeneous Pathway Activation and Drug Response Modelled in Colorectal-Tumor-Derived 3D Cultures. Plos Genet. 15, e1008076. 10.1371/journal.pgen.1008076 30925167 PMC6457557

[B203] Schütte M. Risch T. Abdavi-Azar N. Boehnke K. Schumacher D. Keil M. (2017). Molecular Dissection of Colorectal Cancer in Pre-clinical Models Identifies Biomarkers Predicting Sensitivity to EGFR Inhibitors. Nat. Commun. 8, 14262. 10.1038/ncomms14262 28186126 PMC5309787

[B204] Seghers A.-K. Cuyle P.-J. Van Cutsem E. (2020). Molecular Targeting of a BRAF Mutation in Pancreatic Ductal Adenocarcinoma: Case Report and Literature Review. Targ Oncol. 15, 407–410. 10.1007/s11523-020-00727-9 32495162

[B205] Serasinghe M. N. Wieder S. Y. Renault T. T. Elkholi R. Asciolla J. J. Yao J. L. (2015). Mitochondrial Division Is Requisite to RAS-Induced Transformation and Targeted by Oncogenic MAPK Pathway Inhibitors. Mol. Cel 57, 521–536. 10.1016/j.molcel.2015.01.003 PMC4320323 25658204

[B206] Serra R. W. Fang M. Park S. M. Hutchinson L. Green M. R. (2014). A KRAS-Directed Transcriptional Silencing Pathway that Mediates the CpG Island Methylator Phenotype. Elife 3, e02313. 10.7554/elife.02313 24623306 PMC3949416

[B207] Seufferlein T. Van Laethem J. L. Van Cutsem E. Berlin J. D. Büchler M. Cervantes A. (2014). The Management of Locally Advanced Pancreatic Cancer: European Society of Digestive Oncology (ESDO) Expert Discussion and Recommendations from the 14th ESMO/World Congress on Gastrointestinal Cancer, Barcelona. Ann. Oncol. 25, ii1–ii4. 10.1093/annonc/mdu163

[B208] Shen W. Tao G.-q. Zhang Y. Cai B. Sun J. Tian Z.-q. (2017). TGF-β in Pancreatic Cancer Initiation and Progression: Two Sides of the Same coin. Cell Biosci 7, 39. 10.1186/s13578-017-0168-0 28794854 PMC5545849

[B209] Shimizu T. Tolcher A. W. Papadopoulos K. P. Beeram M. Rasco D. W. Smith L. S. (2012). The Clinical Effect of the Dual-Targeting Strategy Involving PI3K/AKT/mTOR and RAS/MEK/ERK Pathways in Patients with Advanced Cancer. Clin. Cancer Res. 18, 2316–2325. 10.1158/1078-0432.ccr-11-2381 22261800

[B210] Shugang X. Hongfa Y. Jianpeng L. Xu Z. Jingqi F. Xiangxiang L. (2016). Prognostic Value of SMAD4 in Pancreatic Cancer: A Meta-Analysis. Translational Oncol. 9, 1–7. 10.1016/j.tranon.2015.11.007 PMC4800056 26947875

[B211] Siegel R. L. Miller K. D. Fuchs H. E. Jemal A. (2021). Cancer Statistics, 2021. CA A. Cancer J. Clin. 71, 7–33. 10.3322/caac.21654 33433946

[B212] Silverman J. A. Kuhlmann E. T. Zurlo J. Yager J. D. Longnecker D. S. (1990). Expression of C-Myc, C-Raf-1, and C-Ki-Ras in Azaserine-Induced Pancreatic Carcinomas and Growing Pancreas in Rats. Mol. Carcinog. 3, 379–386. 10.1002/mc.2940030610 2278633

[B213] Silvestri A. Schumacher D. Silvestrov M. Schäfer R. Reinhard C. Hoffmann J. (2017). *In Vitro* Three-Dimensional Cell Cultures as Tool for Precision Medicine. Mech. Mol. Carcinogenesis 2, 281–313. 10.1007/978-3-319-53661-310.1007/978-3-319-53661-3_14

[B214] Singh A. Greninger P. Rhodes D. Koopman L. Violette S. Bardeesy N. (2009). A Gene Expression Signature Associated with "K-Ras Addiction" Reveals Regulators of EMT and Tumor Cell Survival. Cancer Cell 15, 489–500. 10.1016/j.ccr.2009.03.022 19477428 PMC2743093

[B215] Singh P. Srinivasan R. Wig J. D. Radotra B. D. (2011). A Study of Smad4, Smad6 and Smad7 in Surgically Resected Samples of Pancreatic Ductal Adenocarcinoma and Their Correlation with Clinicopathological Parameters and Patient Survival. Bmc Res. Notes 4, 560. 10.1186/1756-0500-4-560 22195733 PMC3268768

[B216] Sivaram N. McLaughlin P. A. Han H. V. Petrenko O. Jiang Y.-P. Ballou L. M. (2019). Tumor-intrinsic PIK3CA Represses Tumor Immunogenicity in a Model of Pancreatic Cancer. J. Clin. Invest. 129, 3264–3276. 10.1172/jci123540 31112530 PMC6668699

[B217] Smith D. C. Rosen L. S. Chugh R. Goldman J. W. Xu L. Kapoun A. (2013). First-in-human Evaluation of the Human Monoclonal Antibody Vantictumab (OMP-18R5; Anti-frizzled) Targeting the WNT Pathway in a Phase I Study for Patients with Advanced Solid Tumors. Jco 31, 2540. 10.1200/jco.2013.31.15_suppl.2540

[B218] Soltani A. Torki S. Ghahfarokhi M. S. Jami M. S. Ghatrehsamani M. (2019). Targeting the Phosphoinositide 3-kinase/AKT Pathways by Small Molecules and Natural Compounds as a Therapeutic Approach for Breast Cancer Cells. Mol. Biol. Rep. 46, 4809–4816. 10.1007/s11033-019-04929-x 31313132

[B219] Somerville T. D. D. Xu Y. Miyabayashi K. Tiriac H. Cleary C. R. Maia-Silva D. (2018). TP63-Mediated Enhancer Reprogramming Drives the Squamous Subtype of Pancreatic Ductal Adenocarcinoma. Cel Rep. 25, 1741–1755. e7. 10.1016/j.celrep.2018.10.051 PMC6296757 30428345

[B220] Son J. Lyssiotis C. A. Ying H. Wang X. Hua S. Ligorio M. (2013). Glutamine Supports Pancreatic Cancer Growth through a KRAS-Regulated Metabolic Pathway. Nature 496, 101–105. 10.1038/nature12040 23535601 PMC3656466

[B221] Stephen A. G. Esposito D. Bagni R. K. McCormick F. (2014). Dragging Ras Back in the Ring. Cancer Cell 25, 272–281. 10.1016/j.ccr.2014.02.017 24651010

[B222] Sui H. Pan S.-F. Feng Y. Jin B.-H. Liu X. Zhou L.-H. (2014). Zuo Jin Wan Reverses P-Gp-Mediated Drug-Resistance by Inhibiting Activation of the PI3K/Akt/NF-Κb Pathway. BMC Complement. Altern. Med. 14, 279. 10.1186/1472-6882-14-279 25085593 PMC4288643

[B223] Swinney D. C. Lee J. A. (2020). Recent Advances in Phenotypic Drug Discovery. F1000Res 9, 944. 10.12688/f1000research.25813.1 PMC7431967 32850117

[B224] Tang D. Wu D. Hirao A. Lahti J. M. Liu L. Mazza B. (2002). ERK Activation Mediates Cell Cycle Arrest and Apoptosis after DNA Damage Independently of P53. J. Biol. Chem. 277, 12710–12717. 10.1074/jbc.m111598200 11821415

[B225] Tang Y. Zhang Z. Tang Y. Chen X. Zhou J. (2018). Identification of Potential Target Genes in Pancreatic Ductal Adenocarcinoma by Bioinformatics Analysis. Oncol. Lett. 16, 2453–2461. 10.3892/ol.2018.8912 30013637 PMC6036577

[B226] Tew B. Y. Durand J. K. Bryant K. L. Hayes T. K. Peng S. Tran N. L. (2020). Genome-wide DNA Methylation Analysis of KRAS Mutant Cell Lines. Sci. Rep. 10, 10149. 10.1038/s41598-020-66797-x 32576853 PMC7311523

[B227] Thomas D. A. Massagué J. (2005). TGF-β Directly Targets Cytotoxic T Cell Functions during Tumor Evasion of Immune Surveillance. Cancer Cell 8, 369–380. 10.1016/j.ccr.2005.10.012 16286245

[B228] Thorpe L. M. Yuzugullu H. Zhao J. J. (2015). PI3K in Cancer: Divergent Roles of Isoforms, Modes of Activation and Therapeutic Targeting. Nat. Rev. Cancer 15, 7–24. 10.1038/nrc3860 25533673 PMC4384662

[B229] Tiriac H. Belleau P. Engle D. D. Plenker D. Deschênes A. Somerville T. D. D. (2018). Organoid Profiling Identifies Common Responders to Chemotherapy in Pancreatic Cancer. Cancer Discov. 8, 1112–1129. 10.1158/2159-8290.cd-18-0349 29853643 PMC6125219

[B230] Tolcher A. W. Khan K. Ong M. Banerji U. Papadimitrakopoulou V. Gandara D. R. (2015). Antitumor Activity in RAS-Driven Tumors by Blocking AKT and MEK. Clin. Cancer Res. 21, 739–748. 10.1158/1078-0432.ccr-14-1901 25516890 PMC4335074

[B231] Torii S. Yamamoto T. Tsuchiya Y. Nishida E. (2006). ERK MAP Kinase in G1 Cell Cycle Progression and Cancer. Cancer Sci. 97, 697–702. 10.1111/j.1349-7006.2006.00244.x 16800820 PMC11158792

[B232] Trotta R. Col J. D. Yu J. Ciarlariello D. Thomas B. Zhang X. (2008). TGF-β Utilizes SMAD3 to Inhibit CD16-Mediated IFN-γ Production and Antibody-dependent Cellular Cytotoxicity in Human NK Cells. J. Immunol. 181, 3784–3792. 10.4049/jimmunol.181.6.3784 18768831 PMC2924753

[B233] Tsai S. McOlash L. Palen K. Johnson B. Duris C. Yang Q. (2018). Development of Primary Human Pancreatic Cancer Organoids, Matched Stromal and Immune Cells and 3D Tumor Microenvironment Models. Bmc Cancer 18, 335. 10.1186/s12885-018-4238-4 29587663 PMC5870823

[B234] Tu J. Park S. Yu W. Zhang S. Wu L. Carmon K. (2019). The Most Common RNF43 Mutant G659Vfs41 Is Fully Functional in Inhibiting Wnt Signaling and Unlikely to Play a Role in Tumorigenesis. Sci. Rep., 9, 711382. 10.1101/711382 PMC6898356 31811196

[B235] Tuveson D. Clevers H. (2019). Cancer Modeling Meets Human Organoid Technology. Science 364, 952–955. 10.1126/science.aaw6985 31171691

[B236] Ueki T. Toyota M. Skinner H. Walter K. M. Yeo C. J. Issa J. P. (2001). Identification and Characterization of Differentially Methylated CpG Islands in Pancreatic Carcinoma. Cancer Res. 61, 8540–8546. 11731440

[B237] Ueki T. Toyota M. Sohn T. Yeo C. J. Issa J. P. Hruban R. H. (2000). Hypermethylation of Multiple Genes in Pancreatic Adenocarcinoma. Cancer Res. 60, 1835–1839. 10766168

[B238] Ullrich A. Schlessinger J. (1990). Signal Transduction by Receptors with Tyrosine Kinase Activity. Cell 61, 203–212. 10.1016/0092-8674(90)90801-k 2158859

[B239] Van Cutsem E. van de Velde H. Karasek P. Oettle H. Vervenne W. L. Szawlowski A. (2004). Phase III Trial of Gemcitabine Plus Tipifarnib Compared with Gemcitabine Plus Placebo in Advanced Pancreatic Cancer. Jco 22, 1430–1438. 10.1200/jco.2004.10.112 15084616

[B240] Vander Heiden M. G. Cantley L. C. Thompson C. B. (2009). Understanding the Warburg Effect: The Metabolic Requirements of Cell Proliferation. Science 324, 1029–1033. 10.1126/science.1160809 19460998 PMC2849637

[B241] van de Wetering M. Francies H. E. Francis J. M. Bounova G. Iorio F. Pronk A. (2015). Prospective Derivation of a Living Organoid Biobank of Colorectal Cancer Patients. Cell 161, 933–945. 10.1016/j.cell.2015.03.053 25957691 PMC6428276

[B242] Viale A. Pettazzoni P. Lyssiotis C. A. Ying H. Sánchez N. Marchesini M. (2014). Oncogene Ablation-Resistant Pancreatic Cancer Cells Depend on Mitochondrial Function. Nature 514, 628–632. 10.1038/nature13611 25119024 PMC4376130

[B243] Vincent A. Herman J. Schulick R. Hruban R. H. Goggins M. (2011a). Pancreatic Cancer. The Lancet 378, 607–620. 10.1016/s0140-6736(10)62307-0 PMC3062508 21620466

[B244] Vincent A. Omura N. Hong S.-M. Jaffe A. Eshleman J. Goggins M. (2011b). Genome-Wide Analysis of Promoter Methylation Associated with Gene Expression Profile in Pancreatic Adenocarcinoma. Clin. Cancer Res. 17, 4341–4354. 10.1158/1078-0432.ccr-10-3431 21610144 PMC3131423

[B245] Vlachogiannis G. Hedayat S. Vatsiou A. Jamin Y. Fernández-Mateos J. Khan K. (2018). Patient-derived Organoids Model Treatment Response of Metastatic Gastrointestinal Cancers. Science 359, 920–926. 10.1126/science.aao2774 29472484 PMC6112415

[B246] Von Hoff D. D. Ervin T. Arena F. P. Chiorean E. G. Infante J. Moore M. (2013). Increased Survival in Pancreatic Cancer with Nab-Paclitaxel Plus Gemcitabine. N. Engl. J. Med. 369, 1691–1703. 10.1056/nejmoa1304369 24131140 PMC4631139

[B247] Waddell N. Pajic M. Pajic M. Patch A.-M. Chang D. K. Kassahn K. S. (2015). Whole Genomes Redefine the Mutational Landscape of Pancreatic Cancer. Nature 518, 495–501. 10.1038/nature14169 25719666 PMC4523082

[B248] Walker E. J. Ko A. H. Holly E. A. Bracci P. M. (2015). Statin Use and Risk of Pancreatic Cancer: Results from a Large, Clinic-Based Case-Control Study. Cancer 121, 1287–1294. 10.1002/cncr.29256 25649483 PMC4393339

[B249] Wang J. P. Wu C.-Y. Yeh Y.-C. Shyr Y.-M. Wu Y.-Y. Kuo C.-Y. (2015). Erlotinib Is Effective in Pancreatic Cancer with Epidermal Growth Factor Receptor Mutations: a Randomized, Open-Label, Prospective Trial. Oncotarget 6, 18162–18173. 10.18632/oncotarget.4216 26046796 PMC4627242

[B250] Warshaw A. L. Castillo C. F.-d. (1992). Pancreatic Carcinoma. N. Engl. J. Med. 326, 455–465. 10.1056/nejm199202133260706 1732772

[B251] Wensink G. E. Elias S. G. Mullenders J. Koopman M. Boj S. F. Kranenburg O. W. (2021). Patient-derived Organoids as a Predictive Biomarker for Treatment Response in Cancer Patients. Npj Precis. Onc. 5, 30. 10.1038/s41698-021-00168-1 PMC8042051 33846504

[B252] Wilson E. B. El-Jawhari J. J. Neilson A. L. Hall G. D. Melcher A. A. Meade J. L. (2011). Human Tumour Immune Evasion via TGF-β Blocks NK Cell Activation but Not Survival Allowing Therapeutic Restoration of Anti-tumour Activity. Plos One 6, e22842. 10.1371/journal.pone.0022842 21909397 PMC3167809

[B253] Witkiewicz A. K. Balaji U. Eslinger C. McMillan E. Conway W. Posner B. (2016). Integrated Patient-Derived Models Delineate Individualized Therapeutic Vulnerabilities of Pancreatic Cancer. Cel Rep. 16, 2017–2031. 10.1016/j.celrep.2016.07.023 PMC5287055 27498862

[B254] Witkiewicz A. K. McMillan E. A. Balaji U. Baek G. Lin W.-C. Mansour J. (2015). Whole-exome Sequencing of Pancreatic Cancer Defines Genetic Diversity and Therapeutic Targets. Nat. Commun. 6, 6744. 10.1038/ncomms7744 25855536 PMC4403382

[B255] Wong M. H. Xue A. Julovi S. M. Pavlakis N. Samra J. S. Hugh T. J. (2014). Cotargeting of Epidermal Growth Factor Receptor and PI3K Overcomes PI3K-Akt Oncogenic Dependence in Pancreatic Ductal Adenocarcinoma. Clin. Cancer Res. 20, 4047–4058. 10.1158/1078-0432.ccr-13-3377 24895459

[B256] Wu B. U. Chang J. Jeon C. Y. Pandol S. J. Huang B. Ngor E. W. (2015). Impact of Statin Use on Survival in Patients Undergoing Resection for Early-Stage Pancreatic Cancer. Am. J. Gastroenterol. 110, 1233–1239. 10.1038/ajg.2015.217 26195180 PMC4877304

[B257] Wu C.-Y. C. Carpenter E. S. Takeuchi K. K. Halbrook C. J. Peverley L. V. Bien H. (2014). PI3K Regulation of RAC1 Is Required for KRAS-Induced Pancreatic Tumorigenesis in Mice. Gastroenterology 147, 1405–1416. e7. 10.1053/j.gastro.2014.08.032 25311989 PMC4252806

[B258] Wu D.-j. Jiang Y.-s. He R.-z. Tao L.-y. Yang M.-w. Fu X.-l. (2018). High Expression of WNT7A Predicts Poor Prognosis and Promote Tumor Metastasis in Pancreatic Ductal Adenocarcinoma. Sci. Rep. 8, 15792. 10.1038/s41598-018-34094-3 30361522 PMC6202314

[B259] Wu D.-m. Zhang T. Liu Y.-b. Deng S.-h. Han R. Liu T. (2019). The PAX6-ZEB2 axis Promotes Metastasis and Cisplatin Resistance in Non-small Cell Lung Cancer through PI3K/AKT Signaling. Cel Death Dis 10, 349. 10.1038/s41419-019-1591-4 PMC6483988 31024010

[B260] Xu W. Wang Z. Zhang W. Qian K. Li H. Kong D. (2015). Mutated K-Ras Activates CDK8 to Stimulate the Epithelial-To-Mesenchymal Transition in Pancreatic Cancer in Part via the Wnt/β-Catenin Signaling Pathway. Cancer Lett. 356, 613–627. 10.1016/j.canlet.2014.10.008 25305448

[B261] Yamada H. Sakamoto H. Taira M. Nishimura S. Shimosato Y. Terada M. (2008). Amplifications of Both C-Ki-Ras with a point Mutation and C-Myc in a Primary Pancreatic Cancer and its Metastatic Tumors in Lymph Nodes. Jpn. J. Cancer Res. 77, 370–375. 10.20772/cancersci1985.77.4 3009377

[B262] Yamada S. Fujii T. Shimoyama Y. Kanda M. Nakayama G. Sugimoto H. (2015). SMAD4 Expression Predicts Local Spread and Treatment Failure in Resected Pancreatic Cancer. Pancreas 44, 660–664. 10.1097/mpa.0000000000000315 25760429

[B263] Yang A. Rajeshkumar N. V. Wang X. Yabuuchi S. Alexander B. M. Chu G. C. (2014). Autophagy Is Critical for Pancreatic Tumor Growth and Progression in Tumors with P53 Alterations. Cancer Discov. 4, 905–913. 10.1158/2159-8290.cd-14-0362 24875860 PMC4125497

[B264] Yang S. Wang X. Contino G. Liesa M. Sahin E. Ying H. (2011). Pancreatic Cancers Require Autophagy for Tumor Growth. Genes Develop. 25, 717–729. 10.1101/gad.2016111 21406549 PMC3070934

[B265] Yao Y. Wei D. (2014). Genomic Instability and Cancer. J. Carcinog Mutagen 05, 1000165. 10.4172/2157-2518.1000165 PMC4274643 25541596

[B266] Yap T. A. Bjerke L. Clarke P. A. Workman P. (2015). Drugging PI3K in Cancer: Refining Targets and Therapeutic Strategies. Curr. Opin. Pharmacol. 23, 98–107. 10.1016/j.coph.2015.05.016 26117819 PMC4728196

[B267] Yen I. Shanahan F. Merchant M. Orr C. Hunsaker T. Durk M. (2018). Pharmacological Induction of RAS-GTP Confers RAF Inhibitor Sensitivity in KRAS Mutant Tumors. Cancer Cell 34, 611–625. e7. 10.1016/j.ccell.2018.09.002 30300582

[B268] Ying H. Elpek K. G. Vinjamoori A. Zimmerman S. M. Chu G. C. Yan H. (2011). PTEN Is a Major Tumor Suppressor in Pancreatic Ductal Adenocarcinoma and Regulates an NF-Κb-Cytokine Network. Cancer Discov. 1, 158–169. 10.1158/2159-8290.cd-11-0031 21984975 PMC3186945

[B269] Ying H. Kimmelman A. C. Lyssiotis C. A. Hua S. Chu G. C. Fletcher-Sananikone E. (2012). Oncogenic Kras Maintains Pancreatic Tumors through Regulation of Anabolic Glucose Metabolism. Cell 149, 656–670. 10.1016/j.cell.2012.01.058 22541435 PMC3472002

[B270] Zeitouni D. Pylayeva-Gupta Y. Der C. Bryant K. (2016). KRAS Mutant Pancreatic Cancer: No Lone Path to an Effective Treatment. Cancers 8, 45. 10.3390/cancers8040045 PMC4846854 27096871

[B271] Zeng G. Germinaro M. Micsenyi A. Monga N. K. Bell A. Sood A. (2006). Aberrant Wnt/β-Catenin Signaling in Pancreatic Adenocarcinoma. Neoplasia 8, 279–289. 10.1593/neo.05607 16756720 PMC1600679

[B272] Zhang Y. E. (2009). Non-Smad Pathways in TGF-β Signaling. Cell Res 19, 128–139. 10.1038/cr.2008.328 19114990 PMC2635127

[B273] Zhao M. Mishra L. Deng C.-X. (2018). The Role of TGF-Β/smad4 Signaling in Cancer. Int. J. Biol. Sci. 14, 111–123. 10.7150/ijbs.23230 29483830 PMC5821033

[B274] Zhong Y. Wang Z. Fu B. Pan F. Yachida S. Dhara M. (2011). GATA6 Activates Wnt Signaling in Pancreatic Cancer by Negatively Regulating the Wnt Antagonist Dickkopf-1. Plos One 6, e22129. 10.1371/journal.pone.0022129 21811562 PMC3139620

[B275] Zhu D. D. Zhang J. Deng W. Yip Y. L. Lung H. L. Tsang C. M. (2015). Significance of NF-Κb Activation in Immortalization of Nasopharyngeal Epithelial Cells. Int. J. Cancer 138, 1175–1185. 10.1002/ijc.29850 26370441

